# Affect-Driven Learning of Robot Behaviour for Collaborative Human-Robot Interactions

**DOI:** 10.3389/frobt.2022.717193

**Published:** 2022-02-21

**Authors:** Nikhil Churamani, Pablo Barros, Hatice Gunes, Stefan Wermter

**Affiliations:** ^1^ Department of Computer Science and Technology, University of Cambridge, Cambridge, United Kingdom; ^2^ Cognitive Architecture for Collaborative Technologies (CONTACT) Unit, Istituto Italiano di Tecnologia, Genova, Italy; ^3^ Knowledge Technology, Department of Informatics, University of Hamburg, Hamburg, Germany

**Keywords:** human-robot interaction, multi-modal affect perception, core affect, reinforcement learning, neural networks

## Abstract

Collaborative interactions require social robots to share the users’ perspective on the interactions and adapt to the dynamics of their affective behaviour. Yet, current approaches for affective behaviour generation in robots focus on instantaneous perception to generate a one-to-one mapping between observed human expressions and static robot actions. In this paper, we propose a novel framework for affect-driven behaviour generation in social robots. The framework consists of (i) a hybrid neural model for evaluating facial expressions and speech of the users, forming intrinsic affective representations in the robot, (ii) an *Affective Core*, that employs self-organising neural models to embed behavioural traits like *patience* and *emotional actuation* that modulate the robot’s affective appraisal, and (iii) a Reinforcement Learning model that uses the robot’s appraisal to learn interaction behaviour. We investigate the effect of modelling different affective core dispositions on the affective appraisal and use this affective appraisal as the motivation to generate robot behaviours. For evaluation, we conduct a user study (n = 31) where the NICO robot acts as a *proposer* in the Ultimatum Game. The effect of the robot’s affective core on its negotiation strategy is witnessed by participants, who rank a *patient* robot with *high emotional actuation* higher on *persistence*, while an *impatient* robot with *low emotional actuation* is rated higher on its *generosity* and *altruistic* behaviour.

## 1 Introduction

In collaborative Human-Robot Interaction (HRI) scenarios, where robots need to effectively engage with humans, it is important for them to perceive and understand human behaviour (Breazeal, 2003) in order to attribute context to their interactions. By adopting a shared perspective of their conversations with users, robots can not only improve their understanding of how an individual experiences an interaction but also adapt their own behaviour to match user expectations. Instead of using static behaviour policies that may fail to engage users over continued interactions (Leite et al., 2013), such an adaptive interaction strategy can help improve the overall interaction experience of the users with the robot. To achieve this, much of the current research in Affective Computing and Social Robotics employs perception models that rely on instantaneous (frame-based or using very-short sequences) evaluation of human affective behaviour (see (Sariyanidi et al., 2015; Corneanu et al., 2016) for an overview). Longer context-driven conversations, however, require robots to analyse and understand how human behaviour evolves (Kirby et al., 2010; Baxter et al., 2011), modelling affective representations (Scherer, 2000; Kirby et al., 2010) that can track their affective behaviour over an entire interaction, creating a much more dynamic and robust understanding for the robot.

Depending upon the interaction context, focusing on developing characteristic behavioural tendencies in robots such as composure or emotional stability may influence how they interact with the users, modulating their appraisal as well as their behaviour (Han et al., 2013). The motivation for embedding such behavioural tendencies in robots comes from human-human interactions where specific personality traits of individuals are shown to impact their interactions with others (Cuperman and Ickes, 2009). An individual’s temperament, that is, the inherent inclinations that shape up their behaviour (Rothbart et al., 2000), influences their subjective appraisal of the environment (Thomas et al., 1970) as well as their decision-making (Bandyopadhyay et al., 2013). Additionally, from the early stages of development, human behaviour is seen to be governed by an *affective core* (Emde et al., 1991; Russell, 2003) that develops, initially, as a procedural understanding of their surroundings, and later, to a more cognitive representation that influences human *agency* and *behaviour*. Such intrinsic self-regulatory tendencies acquired as a result of interactions with their environment are essential for human cognitive development and act as anchors for their perception and understanding. Furthermore, individualistic attributes of *temperament*, evolving into *personality*, can be seen as the “basis for dispositions and orientations towards others and the physical world and for shaping the person’s adaptations to that world” (Rothbart et al., 2000).

For social robots, such predispositions (or *perceptual* and *behavioural* tendencies) can be achieved by embedding modulations on their perception and decision-making that impact their behaviour during interactions. Collaborative HRI scenarios also require modelling naturalistic interaction dynamics between humans and robots. Hence, achieving *adaptability*, such that a robot shows an improved and evolving understanding of the dynamics of its interactions with the users becomes a principal objective. Exhibiting such *personal ontogeny* (Robins et al., 2005) also hints at the robot intelligently interacting with users.

In this work, we propose a novel framework for affect-driven learning of interaction behaviours in collaborative HRI settings where the robot’s affective appraisal of user behaviour forms the basis for its learning. Different from existing approaches that mimic the user’s expressions (Churamani et al., 2017; Paiva et al., 2017), here we propose forming evolving representations that help track the users’ expressed affective state allowing for personalised and adaptive interactions with users. These affective representations are modelled as the robot’s *affective memory* (Barros and Wermter, 2017) summarising the affective impact of past interactions with a user, as well as its *mood* (Churamani et al., 2018; Barros et al., 2020), that is, its intrinsic state in response to the user’s behaviour. The mood is further modulated by specifically modelled behavioural inclinations that govern the *affective core* of the robot. For this, we examine the impact of *two* specific attributes; *interaction time* and the *social conditioning* of the robot, to form its *affective core*. The robot’s *mood* is then translated into learning appropriate robot behaviours while negotiating resources with users during human-robot interactions. The different components of the proposed framework can be summarised as the following:

1) Firstly, a deep, multi-channel and hybrid neural model is proposed for robust multi-modal affect perception, evaluating the user’s facial expressions and speech. These evaluations help form the *affective memory* and the intrinsic *mood* of the robot in response to the users’ expressed affective states.
2) We propose modelling the *Affective Core* of the robot using recurrent self-organising neural networks to enforce distinct affective dispositions influencing its *mood.* For this, we consider two specific factors namely, “time perception” as the impact of the duration of an interaction, and the robot’s “social conditioning” or emotional actuation, that is, the intensity of the interaction. Both these influences are used as modulations on the robot’s perception, resulting in significantly different *mood* responses towards the users’ affective behaviour.
3) Finally, translating the robot’s *mood*, that is, its intrinsic affective response towards the user into interaction behaviour, we map this *mood* onto the state-space for the robot to learn optimal negotiating behaviour in the Ultimatum Game (Harsanyi, 1961; Güth et al., 1982). The robot and the user negotiate a split of resources amongst themselves, underlining the expectations from robots in collaborative HRI scenarios, particularly concerning adaptability and naturalistic interaction. An actor-critic-based (Lillicrap et al., 2015) Reinforcement Learning (RL) model is employed that learns to negotiate resources with users, adapting based on their affective responses towards the robot’s offers.

## 2 Related Work

The affective impact of one’s interactions with others plays an important role in human cognition (Jeon, 2017). The *core affect* in an individual forms a neurophysiological state (Russell, 2003) resulting from the interplay between the valence of an experience and the emotional arousal it invokes. This influences how people perceive situations and regulates their responses. Understanding the evolution of human affective behaviour enables us to emulate such characteristics in social robots. It allows robots to ground intrinsic models of affect to improve their interaction capabilities. In this section, we present a brief overview on multi-modal affect perception (Section 2.1), the representation of affect as an intrinsic attribute (Section 2.2), and behaviour synthesis (Section 2.3) in social robots discussing different existing frameworks that use affective appraisal as the basis for modelling robot behaviour in HRI scenarios.

### 2.1 Multi-Modal Affect Perception

Humans interact with each other using different verbal and non-verbal cues such as facial expressions, body gestures and speech. For social robots, analysing user behaviour across multiple modalities improves their perception capabilities. Furthermore, in case of masked perception or conflicts, this additional information may enable the robot to resolve conflicts (Parisi et al., 2017a). Although various *outward* signals (Gunes et al., 2011) can be observed to model affect perception in agents, facial expressions and human speech are the predominantly used modalities to evaluate human affective behaviour in HRI settings (Spezialetti et al., 2020).

Facial expressions can be categorised into several categories (Ekman and Friesen, 1971) or represented on a dimensional scale (Yannakakis et al., 2017). Traditionally, computational models have used shape-based, spectral or histogram-based analysis for affect perception (see (Zeng et al., 2009; Sariyanidi et al., 2015; Corneanu et al., 2016) for a detailed analysis), but more recently, deep learning has enhanced the performance of Facial Expression Recognition (FER) models by reducing the dependency on the choice of features and instead, learning directly from the data (Kollias and Zafeiriou, 2018; Li and Deng, 2020). Although these work well in clean and noise-free environments, spontaneous emotion recognition in less controlled settings is still a challenge (Sariyanidi et al., 2015). Thus, the focus has now shifted towards developing techniques that are able to recognise facial expressions in real-world conditions (Kossaifi et al., 2017; Zafeiriou et al., 2017), robust to movements of the observed person, noisy environments and occlusions (Zen et al., 2016).

Analysing human speech, either by processing spoken words to extract the sentiment behind them or understanding speech intonations, offers another potent way of evaluating human affective expression during interactions. While spoken words convey meaning, *paralinguistic* cues enhance a conversation by highlighting the affective motivations behind these spoken words (Gunes and Pantic, 2010). Despite providing information about the context and intent (Whissell, 1989) in an interaction, it is difficult to deduce the affective state of an individual using only spoken words (Furnas et al., 1987). Extracting spectral and prosodic representations can help better analyse affective undertones in speech. Different studies on Speech Emotion Recognition (SER) (see (Schuller, 2018) for an overview) make use of representations such as Mel-Frequency Cepstral Coefficients (MFCC) or features like *pitch* and *energy* to evaluate affective expression. More recently (deep) learning is employed to extract relevant features directly from the raw audio signals (Keren and Schuller, 2016; Tzirakis et al., 2017).

Most of the current approaches (Poria et al., 2017; Tzirakis et al., 2017; Spezialetti et al., 2020) combine face and auditory modalities to determine the affective state expressed by an individual. This combination can either be achieved using weighted averaging or majority voting (Schels et al., 2013) from individual modalities (Busso et al., 2004) or feature-based sensor fusion (Kahou et al., 2016; Tzirakis et al., 2017) and deep learning (Poria et al., 2017).

### 2.2 Representing Affect in Social Agents

For long-term adaptation, it is important that robots not only recognise users’ affective expressions but also model *continually* evolving intrinsic representations (Paiva et al., 2014) that track human affective behaviour. Kirby et al. (2010) explore slow-evolving affect models such as *moods* and *attitudes* that consider the personal history and the environment to estimate an affective state for the robot in response to the users. Barros et al. (Barros et al., 2020) also propose the formation of an intrinsic *mood* that uses an *affective memory* (Barros and Wermter, 2017) of the individual as an influence over the perception model. The WASABI model (Becker-Asano and Wachsmuth, 2009) represents the intrinsic state of the robot on a *PAD*-scale that adapts based on the agent’s interactions with the user. In the SAIBA framework (Le et al., 2011), the agent’s intrinsic state is modelled using mark-up languages that model intent in the robot and use it to generate corresponding agent behaviour. The (DE)SIRE framework (Lim et al., 2012) represents this intrinsic affect as a vector in a 4-d space for the robot which is then mapped to corresponding expressions across different modalities. Although all these approaches are able to provide the necessary basis for modelling affective and behavioural dispositions in agents, they require careful initialisation, across *n*-dimensional vector-spaces, to result in the desired effect. It will be beneficial if these intrinsic representations could be learnt dynamically by the agent as a result of its interactions.

### 2.3 Behaviour Synthesis in Social Agents

Recent works on behaviour learning in social agents investigate the role of affect as a motivation to interact with their environment. Such strategies may include affective modulation on the computation of the reward function where explicit feedback from the user is shown to speed up learning (Broekens, 2007). Alternatively, affective appraisal can be viewed as an inherent quality of the robot, motivating it to interact with its environment (Han et al., 2013; Moerland et al., 2018). Affect is modelled as an evaluation of physiological changes (changing battery level or motor temperatures) that occur in the robot, with their behaviour influenced by *homeostatic* drives that lead towards a stable internal state (Konidaris and Barto, 2006). Other approaches examine different cues such as *novelty* and the *relevance* of an action to the task to appraise the robot’s performance (Sequeira et al., 2011). In the case of *value-based* approaches (Jacobs et al., 2014), the state-space of the robot is mapped onto different affective states and the value of any state represents the affective experience of the robot in that state. Reward-based approaches, on the other hand, consider *temporal changes* in the reward or the reward itself as the basis of the robot experiencing different affective states (Ahn and Picard, 2005).

We propose using the robot’s affective perception, modulated by its past experiences with the users as well as specific affective dispositions forming its *affective core*, to govern its responses towards the users during interactions. Using the robots’ *mood* as their subjective evaluation of an interaction, we aim to embed robots with adaptive interaction capabilities that learn task-specific behaviour policies by developing a shared perception of the interaction as well as the users’ expectations from the robots.

## 3 The Proposed Framework

**FIGURE 1:**
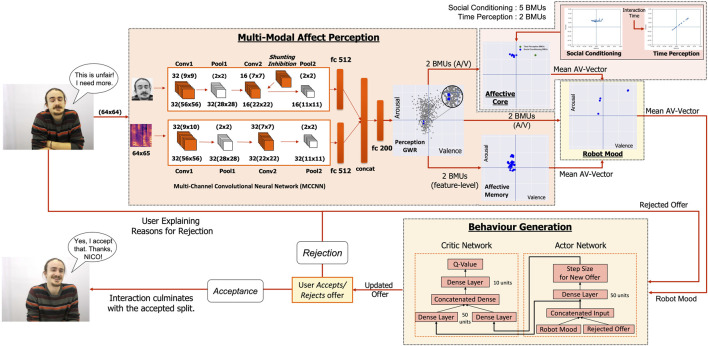
Proposed Framework: Multi-Modal Affect Perception of the robot combines facial and auditory features using the MCCNN. The Perception-GWR creates feature prototypes with the BMUs encoding arousal-valence while repeated interactions form the Affective Memory. The Affective Core models affective dispositions in the robot, resulting from its social conditioning and time perception. Current perception, affective memory and the affective core influence Mood formation which is used by the Behaviour Generation model to learn negotiating behaviour in the Ultimatum Game.

In this paper, we propose a novel framework that combines multi-modal affect perception in robots with learning adaptive interaction behaviour in collaborative HRI settings. The proposed framework consists of four main components (see Figure 1); (i) a multi-modal affect perception model (see Section 3.1) that is used to analyse the users’ facial expressions and speech and continuously track the user’s affective behaviour, (ii) the *affective core* of the robot (see Section 3.2) that models affective dispositions governing the perception as well as the behaviour of the robot, (iii) the intrinsic *mood* of the robot (see Section 3.3) that describes how the robots affect perception and its *affective core* impact its intrinsic responses towards the user, and finally (iv) an RL-based (Lillicrap et al., 2015) behavioural learning model (see Section 3.4) that translates the robot’s intrinsic *mood* into effective negotiating behaviours while interacting with the users in the Ultimatum Game (Harsanyi, 1961; Güth et al., 1982) scenario.

### 3.1 Multi-Modal Affect Perception

The affect perception model, adapted from (Churamani et al., 2018; Barros et al., 2020), consists of three components, namely the Multi-Channel Convolutional Neural Network (MCCNN) network (Barros and Wermter, 2016) for multi-modal feature extraction and fusion, the Perception- Growing-When-Required (GWR) Neural Network for prototyping the extracted features to improve the robustness of the model to changing lighting conditions and variance within an individual’s expressions, and the *affective memory* GWR Neural Network (Barros and Wermter, 2017) that evaluates how the affective state of the user evolves during an interaction (see Figure 1).

#### 3.1.1 The MCCNN Network

The MCCNN (Barros and Wermter, 2016; Churamani et al., 2018) consists of two separate channels for processing facial and auditory information and then combines the learnt features into a combined dense representation. Rather than using categorical labels for classification, the model is adapted to represent affect in the form of *arousal* and *valence* dimensions.

The face channel takes a (64 × 64) greyscaled *mean-face* image from every 12 frames (considering a 500 milliseconds window) recorded at 25 FPS to reduce spurious effects caused due to camera flickers and refresh-rates. It consists of 2 convolutional (*conv*) layers, each followed by (2 × 2) max-pooling. The first layer performs (9 × 9) convolutions while the second layer consists of (7 × 7) filters using *shunting inhibition* (Frégnac et al., 2003) to obtain filters robust to geometric distortions. The *conv* layers are followed by a fully-connected (FC) layer consisting of 512 units.

The audio channel uses Mel-spectrograms^1^ computed for every 500 milliseconds of the audio signal, re-sampled to 16 kHz and *pre-emphasised* to amplify the high frequencies and balancing the frequency spectrum to improve the Signal-to-Noise ratio (Picone, 1993). A frequency resolution of 1,024 Hz is used, with a *Hamming window* of 10 ms, generating Mel-spectrograms consisting of 64 bins with 65 descriptors each. The audio channel consists of 2 *conv* layers with a filter size of (9 × 10) and (7 × 7) each followed by (2 × 2) max-pooling. The conv layers are followed by a FC layer with 512 units.

The FC layers from both the face and audio channels are concatenated into a single FC layer consisting of 1,024 units and connected to another FC layer consisting of 200 units. This enables the network to be trained to extract features that are able to predict arousal and valence values by combining the two modalities. For training the MCCNN (see Section 4.1.1), the 200-d FC layer is connected to a 2-unit linear activation-based classifier layer to predict *arousal* and *valence* values. Once trained, the classifier layer is removed and the 200-d feature vectors are used to train the rest of the model. The trained MCCNN-classifier layer is used again to classify the feature representations learned by the Perception-GWR into arousal-valence values. The hyper-parameters for MCCNN, that is, the number of filters and filter sizes for each layer of the face and audio-channel as well as the FC layer sizes are optimised using the Hyperopt^2^ python library. More details can be found in Supplementary Material.

#### 3.1.2 Perception-GWR

Even though the MCCNN-classifier layer can be used directly to predict the affective state of the user (arousal and valence), each individual expresses differently and this variance may result in different outputs for the same affective state expressed by different users. Thus, to allow for a more robust approach, it is beneficial to adopt a developmental view on affect perception that can account for the variance with which users express their affective state (Barros and Wermter, 2016; Churamani et al., 2018). We achieve this by using a Growing-When-Required (GWR) network (Marsland et al., 2002) that incrementally prototypes feature representations, extracted by the MCCNN model as the agent observes the users, accounting for the variance in audio-visual stimuli (Barros et al., 2020). The GWR implements a *Competitive Hebbian-Learning-based* mechanism (Martinetz, 1993) for adapting the topology of the network, depending upon the input features. New neurons are added to the GWR whenever the existing neurons are not enough to represent the input adequately, that is, the activation of the existing neurons fall below a given *activation threshold*. This dynamic growth of the model results in an optimum mapping of the input space to the learnt feature representations, starting from two randomly assigned neurons with new neurons added to adequately represent the input space with adaptive fine-tuning as the learning progresses.

The Perception-GWR learns, in an unsupervised manner (see Section 4.1.2), using the multi-modal features extracted as the 200 unit FC layer from the pre-trained MCCNN model. Thus, rather than considering the output of the MCCNN-classifier, we extract learnt feature prototypes from the Perception-GWR by taking the two *winner* neurons or Best Matching Units (BMUs) (Marsland et al., 2002) closest to the input, that is, the neurons with weights closest to the input feature vector in a Euclidean space. These feature prototypes offer a much more effective way to represent learnt feature representations, robust to variation in individual expression and/or changing lighting and environmental conditions (Barros et al., 2020). The winner neurons are used to predict the encoded arousal-valence values using the pre-trained MCCNN-classifier layer.

#### 3.1.3 Affective Memory

To encode how the affective behaviour of the user evolves during an entire interaction, apart from analysing the users’ expressed affective state, the robot also needs to account for past encounters with them, forming a memory model that grows and adapts over time. Such a personalised *affective memory* (Barros and Wermter, 2017; Barros et al., 2019) (see Figure 1), developing as the robot interacts with the user, forms an expectation model for the robot that can reduce the impact of spurious effects in perception due to misclassifications or noise. As users interact with the robot, BMUs or *winner neurons* from the Perception-GWR model, that is, 200 − d multi-modal feature prototypes for every 500 milliseconds of audio-visual input, are used to train the robot’s *affective memory* (see Section 4.1.2). This memory is modelled using a Gamma-GWR network (Parisi et al., 2017b) (explained in detail in Section 3.2) consisting of neurons with recurrent connections to remember the effect of past interactions.

### 3.2 Modelling the Affective Core of the Robot

The *Affective Core* in humans acts as an affective disposition, not just contributing towards their affective appraisal, but also governing their behaviour (Emde et al., 1991). Similarly, an affective core for a robot can be used as the basis for inherent affective dispositions that may influence its perception and behaviour. For this, we propose the use of Recurrent Gamma-GWR models (Parisi et al., 2017b), equipped with a Gamma-context memory (de Vries and Principe, 1992), for modelling the *affective core* of the robot. Yet, rather than focusing on the temporal evolution of an expression, for example, *onset* to *offset* for a facial expression (Sariyanidi et al., 2015), we focus on tracking the evolution of the overall affective behaviour over successive time-steps. Thus, instead of evaluating temporal dynamics of user behaviour at the feature-level, the encoded arousal-valence values obtained by classifying the feature prototypes resulting from the Perception-GWR model are examined over the entire duration of the interaction. To account for such temporal dynamics, each neuron is equipped with a fixed number of context descriptors which increase the temporal resolution of the model. The Gamma-GWR model (Parisi et al., 2017b) is equipped with recurrent neurons that use *Gamma-filtering* (de Vries and Principe, 1992) for representing such temporal characteristics of input data. The learning rule and activation functions for the GWR model (Marsland et al., 2002) are modified to account for activation of the neurons from the previous ***K*** (number of *Gamma* filters) time-steps. The BMU or winner neuron *b* is computed as follows:

$$b=\underset{i}{\mathrm{arg min}}\left\{d_{i}\right\},$$ (1)

where *d*_*i*_ is the distance of the neuron *i* from the data-point. The activation takes into account both the distance between the input and the weights at the current time-step as well as uses the context activation over the last *K* gamma filters:

$$d_{i}=\alpha_{w}\;.\;\|x\left(t\right)-w_{i}{\|}^{2}\;+\;\overset{K}{\underset{k=1}{\sum }}\;\alpha_{k}\;.\;\|C_{k}\left(t\right)-c_{i}^{k}{\|}^{2},$$ (2)

where *x*(*t*) represents the current input (in this case, the 200-d feature prototype for the Perception-GWR and the *affective memory*, and the encoded 2-d arousal-valence value for the *affective core*), *w*_*i*_ represents the weight vector of the *i*th neuron, *α*_*w*_ and *α*_*k*_ are constants influencing the modulations from past activation and the current input, $C=\left[c_{1}^{i},c_{2}^{i},\ldots ,c_{k}^{i}\right]$ is the set of context vectors for the *i*th neuron with *k* = 1, 2, *…* , *K* being the Gamma filter order. Global context *C*_*k*_(*t*) is given as:

$$C_{k}\left(t\right)=\beta \;.\;w_{b\left(t-1\right)}\;+\;\left(1-\beta \right)\;.\;c_{b\left(t-1\right)}^{k-1}$$ (3)

where *β* controls the influence of the previous activation on the current processing of input, *b* (*t* − 1) is the winner neuron from the previous time-step and $c_{b\left(t-1\right)}^{0}\equiv w_{b\left(t-1\right)}$ .

Once the BMU is selected, the weight of the winning neuron and the context vectors are updated as follows:

$$\Delta w_{i}=\epsilon_{i}\;.\;\eta_{i}\;.\;\left(x\left(t\right)-w_{i}\right),$$ (4)

$$\Delta c_{i}^{k}=\epsilon_{i}\;.\;\eta_{i}\;.\;\left(C_{k}\left(t\right)-c_{i}^{k}\right),$$ (5)

where *ϵ*_*i*_ is the learning rate that modulates the updates and does not decay over time. The firing counter *η*_*i*_, on the other hand, is used to modulate learning (Marsland et al., 2002). It is initialised to 1 (*η*_0_ = 1) and decreased according to the following rule:

$$\Delta \eta_{i}=\tau_{i}\;.\;\kappa \;.\;\left(1-\eta_{i}\right)-\tau_{i}$$ (6)

where constants *κ* and *τ*_*i*_ control decay curve behaviour.

We explore the influence of two factors, namely *time perception* and the *social conditioning* of the robot, forming the *affective core* of the robot (see Figure 1). While time perception refers to how the robot is impacted by the duration of an interaction, social conditioning accounts for the *acculturation* or emotional actuation of the robot as a result of its repeated interactions with affective stimuli. These qualities, amongst others, are also found to have an influence on the temperament and personality formation in infants (Rothbart et al., 2000) resulting from engagement with caregivers.

#### 3.2.1 Interaction Time Perception

**FIGURE 2:**
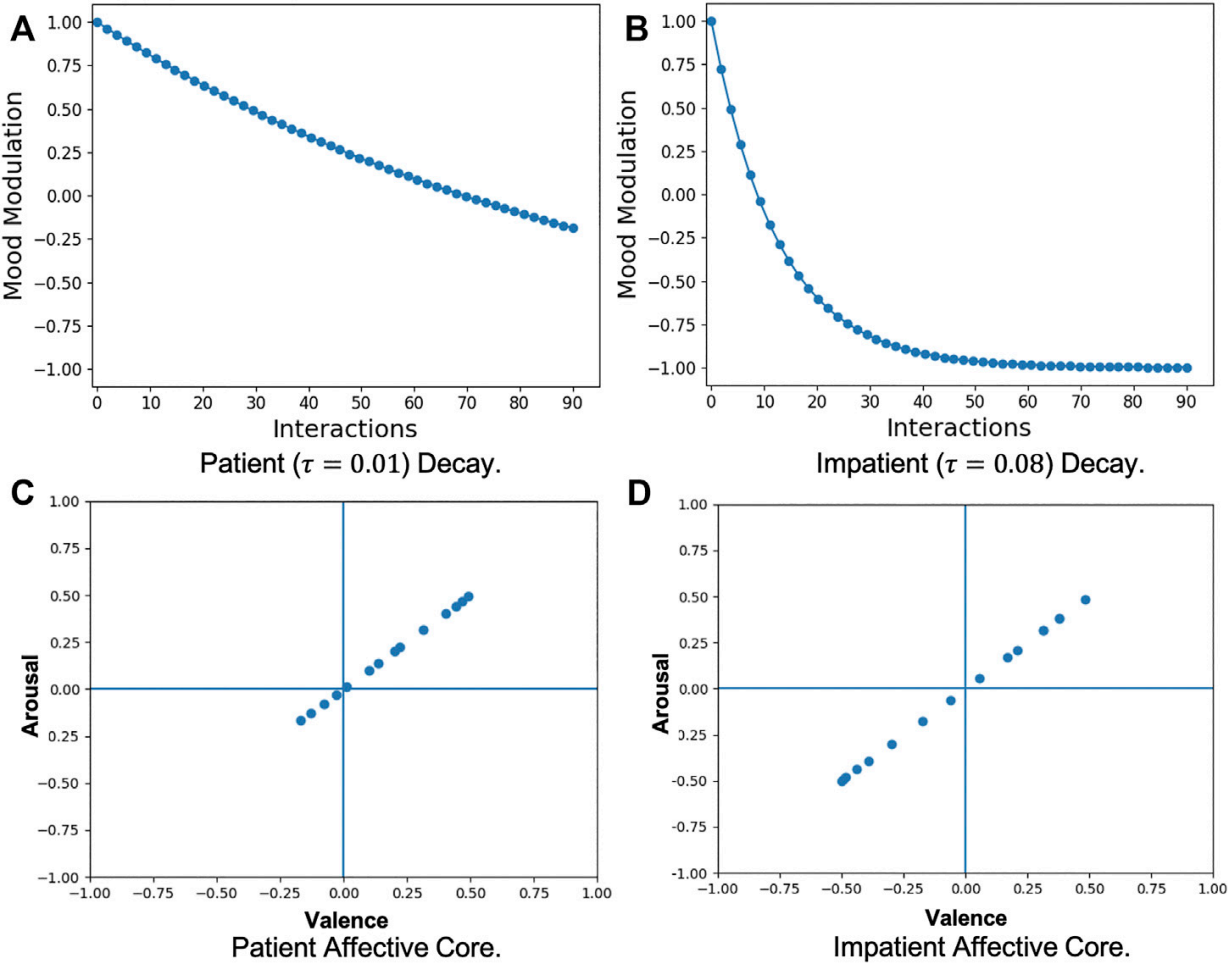
Patient **(A)** and Impatient **(B)** Mood Modulation results in Affective Cores encoding corresponding Patient **(C)** and Impatient **(D)** Time Perception for the agent.

As the robot interacts with users, it forms an intrinsic affective response (the *mood*) towards them based on its perception of user behaviour. This perception can be influenced by the duration of the interaction based on whether the robot has a *patient* or *impatient* affective disposition. Starting from the same initial affective state every time, the robot, given its inherent time perception, tries to maintain its intrinsic state for the entire duration of the interaction with a user. Assuming that the robot’s *mood* develops only as a result of the *duration of an interaction*, a *core affect* based on interaction time perception can be modelled in the robot. For this, we use a modulation function (*y* = exp (−*τt*)) that impacts how the robot evaluates its interactions with the user at any given time. The affective impact of the current perception of the robot, depicted by *x*(*t*) in Eq. 2, is replaced by *y*(*t*) = exp (−*τ*.*x*(*t*)) to model the *core affect* based on the interaction time. With no external input provided, that is, if *x*(*t*) is used to represent only the current mood of the robot, this will result in a *decay* in the robot intrinsic *mood* over time, which is used to train the Patient or Impatient Affective Core for the robot. For simulating *patience*, this decay is modelled to be slow and gradual (*τ* = 0.01), such that the robot maintains its affective state for a longer duration while in case of *impatient* time perception, this decay is rapid (*τ* = 0.08). The decay function dynamics can be seen in Figure 2A,B, respectively. The empirical choice of *τ* values assures smooth decay curves over a minimum of 90 time-steps. These values can be adapted as per the desired impact of time perception in the robot.

Given a *patient* or *impatient* modulation, an *affective core* bias is modelled using the Gamma-GWR model. The initial state of the robot is set to a mean positively excited state with arousal and valence values of (0.5, 0.5) and then modulated over time in the absence of any external input. At each time-step, the Gamma-GWR model receives this modulated input state and forms intrinsic prototypes following the process described in Eqs. 1–6. This dynamically models the robot’s intrinsic state at different time-steps, forming a time perception bias that encodes a *patient* (Figure 2C) or *impatient* (Figure 2D) affective disposition.

#### 3.2.2 Social Conditioning

In their growing years, the interactions with care-givers and their surroundings, in general, have a lasting impact on the personality and temperamental development of humans (Rothbart et al., 2000). Their ability to apprehend and appraise their environments is influenced by such interaction experiences, forming anchors for their affective appraisal. Such a *social conditioning* impacts their personality and behaviour in the long run and has a huge impact on how they interact with others. Such a form of *social conditioning* can also be used to formulate anchors for a robot’s affective appraisal. The robot, through continued and repeated interaction with affective stimuli, can get *acculturated*, developing affective dispositions central to its *personality* and *temperament*. Such a conditioning can be either *excitatory* (high-arousal), amplifying the impact of its perception, or *inhibitory* (low-arousal), diminishing it.

**FIGURE 3:**
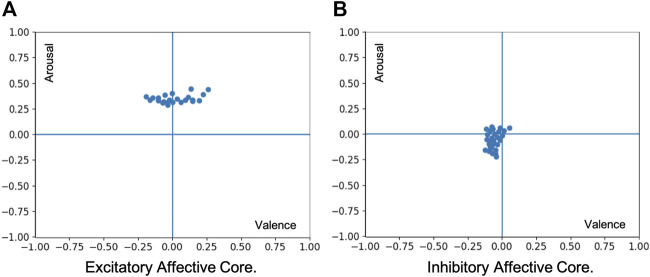
Affective Cores encoding Excitatory or High-arousal **(A)** and Inhibitory or Low-arousal **(B)** modulation to form the Social Conditioning of the agent.

To model such a *core affect*, we present the model with videos encoding different emotional intensities (see Section 4.2 for details). To model an excitatory effect, videos encoding high arousal are used whereas, for the inhibitory effect, low arousal videos are used. This is done to simulate affective acculturation in the robot so as to model contrasting modulations on the robot’s intrinsic *mood*. The videos are processed using the MCCNN-Perception-GWR model where BMUs from the Perception-GWR, for every 500 milliseconds of audio-visual input, are classified (using the MCCNN-classifier) into the corresponding arousal (A) and valence (V) values they encode and prototyped using a Gamma-GWR model. The resultant, prototypical, *excitatory* core affect (Figure 3A) represents only high-arousal information (*A* > 0.3), while the *inhibitory* core affect (Figure 3B) encodes only low-arousal information (*A* < 0.05). As a result, when this *affective core* modulation is applied to the robot’s affective perception, the resultant impact on its intrinsic *mood* is either excitatory (high-arousal) or inhibitory (low-arousal) based on the social conditioning of the robot.

### 3.3 Mood Formation for the Robot

The *mood* of the robot constitutes its intrinsic affective response towards the user during interactions. Such an affective response should take into account several intrinsic (robot-centric) as well as extrinsic (user or context-centric) attributes to evaluate the robot’s interactions with a user. In this work, we propose the robot to use its multi-modal perception to evaluate the user’s current affective state, modulated by its *affective memory* of the user, providing context. Additionally, this affective response is driven by the robot’s inherent *affective core* dispositions, that is, social conditioning and time-perception, defining how the robot experiences an interaction. Thus, *mood* formation in the robot (see Figure 1) accumulates several contributing factors that may impact the affective appraisal of the robot during interactions and forms the basis for the robot’s behaviour in response to these factors.

We model the robot’s *mood* as a Gamma-GWR (Parisi et al., 2017b) (following Eqs. 1–6) that describes the intrinsic response of the robot based on how it experiences an interaction. The *mood* is modelled in an *online* and *continuous* manner not only considering the current behaviour of the user (*perception*), modulated by past experiences (*affective memory*), but also the robot’s *affective core* that governs whether the robot over or under-estimates the affective impact of its perception. For this, multi-modal feature-representations of the user behaviour are extracted every 500 ms using the MCCNN model and the 2 BMUs from the Perception-GWR are computed as a representation of the users’ current behaviour. These feature-representations are passed to the *affective memory* model updating the robot’s experiences with a particular user. To encode the robot’s perception in terms of the arousal-valence values it represents, the Perception-GWR BMUs as well as the *affective memory* neurons are passed to the MCCNN-classifier to encode them into the arousal-valence values they represent. All the neurons from the *affective memory* are encoded into a mean arousal-valence value for the entire GWR memory, representing the *net affect* of past experiences with a user. The encoded Perception-GWR BMUs are also passed to the social-conditioning *affective core* (excitatory or inhibitory) and five BMUs are chosen, representing the social conditioning of the robot. To encode the impact of time-perception, following the respective modulation (see Section 3.2.1), 2 BMUs from the time-perception *affective core* (patient or impatient) are chosen. Since the social conditioning *affective core* modulates the current perception directly, more weight is given to it by selecting a higher number of BMUs to flood the *mood* input with the corresponding effect. All these inputs, that is, (i) the affect-encoded Perception-GWR BMUs and the mean *affective memory* arousal-valence value representing the agent’s perception and (ii) the social conditioning and time-perception *affective core* modulations are processed *asynchronously* to allow for the evolution of the robot’s *mood* even when they are sparsely available. The resultant mood of the robot, at any given time, is computed as the mean arousal-valence value for all the neurons in the mood Gamma-GWR model. The robot thus forms an organic affective response towards the user rather than merely mimicking them as the different *affective core* biases in the robot result in the same stimulus being evaluated differently. For example, a *patient* robot with an *excitatory* conditioning is seen to retain its positive mood for longer, even if it receives a series of negative inputs (see Section 4.2 for a detailed analysis). This is important as it can be used to integrate different affective and behavioural dispositions in the robot, with different combinations of the *affective core* influences expected to impact the robot *mood*, and thus its behaviour towards the user, differently.

### 3.4 Behavioural Learning for the Robot

The intrinsic *mood* of the robot forms the affective response of the robot towards the user during an interaction. This *mood* can be used as the basis to learn task or context-specific behaviours, intrinsically modulated by the *affective core* of the robot. In this work, we explore the Ultimatum Game (Harsanyi, 1961; Güth et al., 1982) to embed different negotiating behaviours in the robot using its intrinsic *mood*, modulated by its *affective core*. We propose, a Deep Deterministic Policy Gradients (DDPG)-based actor-critic model (Lillicrap et al., 2015) that learns to interact with human participants, incorporating the robot’s *mood*, both in the state-value function as well as in the reward received by the robot. The proposed model aims to evaluate how the robot, given its intrinsic mood, can learn to successfully negotiate resources with human participants. Furthermore, evaluation of the robot by the participants under different *affective core* conditions can highlight the contribution of modelling specific affective and behavioural dispositions in the robot towards its negotiation capabilities.

#### 3.4.1 The Interaction Scenario: The Ultimatum Game

**FIGURE 4:**
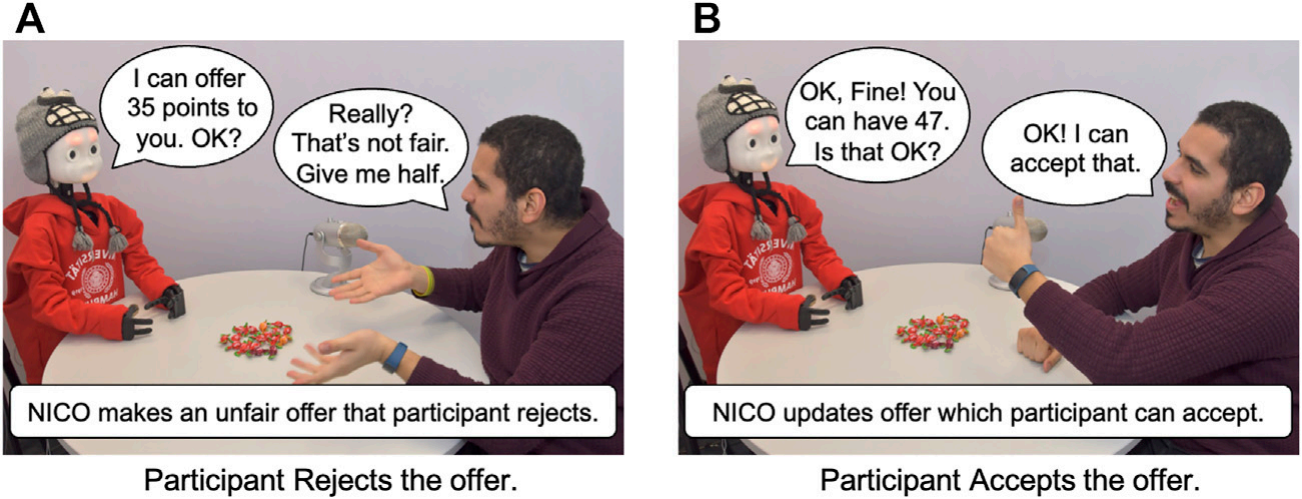
Participant and the NICO robot negotiating in the Ultimatum Game scenario.

The traditional design for the Ultimatum Game (Harsanyi, 1961; Güth et al., 1982) involves two participants namely, a *proposer* and a *respondent*, negotiating a split of resources (usually money). The *proposer* offers a split, based on which the *respondent* either *accepts* or *rejects* the offer. Only if the offer is accepted, resources are shared as per the agreed split. We extend this design by incorporating a “continuous” negotiation between the participants (see Figure 4) and the Neuro-Inspired Companion (NICO) robot (Kerzel et al., 2017) acting as the *proposer*. A similar negotiating strategy is proposed in the Rubinstein alternate-offers bargaining game (Rubinstein, 1982) with the key difference being that both the players in the game take turns as the proposer, proposing splits of the resources until one of them accepts the other’s offer. In this work, however, we enforce the NICO robot to be the proposer in all the negotiation rounds, making our implementation different from the Rubinstein alternate-offers bargaining game. NICO and each participant are given 100 points that can be exchanged for 20 *bonbons*, with every five points fetching them one bonbon. Bonbons are used to give a visual motivation for the negotiation. As NICO makes offers to the respondent, if they accept the offer, the interaction culminates with both receiving the agreed split. In case of a rejection, NICO asks the participants for reasons for their rejection and based on their affective responses, it appraises their affective state as an evaluation of the offer, eliciting a change in its own mood. This mood (or change in the mood over successive negotiations) is used to update its offer in a way that the participant may accept the new offer, without NICO losing a lot of points. The participant and NICO thus negotiate a split of the 100 points with NICO updating its offer upon each rejection. To assure that negotiations come to a conclusion, the negotiation is aborted with no one getting any points after the participant rejects 20 consecutive offers. The participants are requested to communicate clearly using facial expressions and speech, expressing their affective response to NICO’s offers.

#### 3.4.2 Learning Negotiating Behaviour

While negotiating with the participants, the intrinsic *mood* of the robot after each rejection, concatenated with the rejected offer value, is mapped to the state-space of the robot to generate actions in the form of increments or decrements on the previous offer. This results in a continuous, high-dimensional action-space making the use of traditional *Q*-learning approaches difficult as they become intractable in such high-dimensional spaces (Lillicrap et al., 2015). Also, it is desirable that these updates to the offer are not modelled as fixed increments or decrements to enable a more naturalistic negotiation between participants and NICO. Thus, int this work we employ a DDPG-based actor-critic model (Lillicrap et al., 2015) to learn an optimal negotiating behaviour that can update the offers made to the *respondents* based on the robot’s affective appraisal of their responses.

**FIGURE 5:**
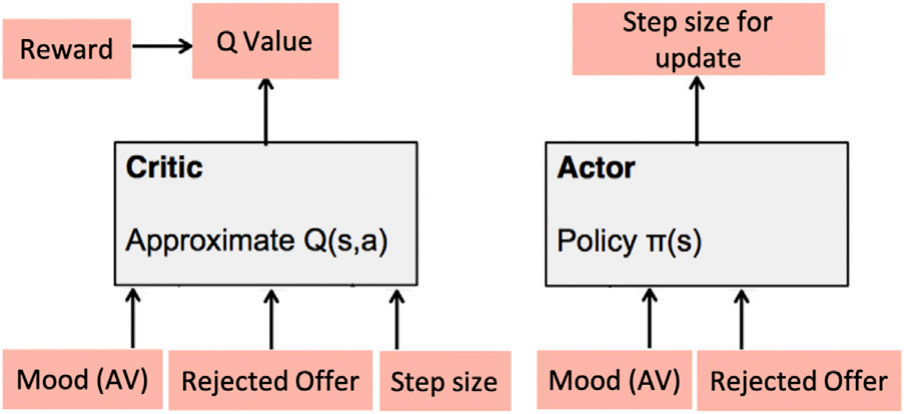
Actor-Critic model for Learning Robot behaviour.

The model consists of two separate networks (see Figure 5) for the *actor* and the *critic*, respectively. The actor network takes the robot’s current mood (mean arousal-valence vector computed from all the neurons of the mood Gamma-GWR model, see Section 3.3) as well as the previously rejected offer^3^ as inputs and concatenates them into a single 4-tuple representing the state of the robot. This state is passed to the *actor*, predicting a real-valued update step-size over the previous offer. The *critic* network takes the current state 4-tuple for the robot as well as the actor-generated update step-size value as inputs to evaluate the actor’s “performance,” predicting a *Q*-value ∈R for the state-action pair. This predicted *Q*-value and the reward received by the robot (see Section 4.3 for details) are used to update both the critic and the actor (Lillicrap et al., 2015).

The actor and critic are modelled as Multilayer Perceptron (MLP) networks (see Figure 1). The input 4-tuple for the actor is connected to an FC layer consisting of 50 units which is further connected to an output neuron, predicting real-valued updates on the offer. For the critic, the state 4 − tuple and the predicted update value are connected to individual FC layers of 50 units each. These FC layers are then concatenated and connected to another FC layer of 10 units combining the representations. Finally, a single output neuron predicts the *Q*-value ∈R for the state-action pair. The hyper-parameters for the DDPG-based actor-critic model are detailed in Table. 1.

**TABLE 1:** Training Parameters for the DDPG algorithm learning to negotiate in the Ultimatum Game.

Parameter	Value
Batch size	10
Replay Buffer Size	100
Actor Learning Rate	0.001
Critic Learning Rate	0.005
Discount Factor (*γ*)	0.9
Soft-update Rate (*τ*)	0.001

## 4 Training and Evaluation

### 4.1 Multi-Modal Affect Perception

To evaluate the multi-modal affect perception model employed in the proposed framework, we train and evaluate the different components separately to ensure that the model is able to extract meaningful feature representations than can be used for training the rest of the framework.

#### 4.1.1 The MCCNN Network

Training a multi-modal continuous affect perception model requires datasets that provide good quality samples for both vision and speech modalities with continuous arousal-valence annotations. Most of the available multi-modal datasets rely on the visual information as the dominant modality deciding affective labels. This is seen in the Aff-Wild (Zafeiriou et al., 2017) and AFEW-VA (Kossaifi et al., 2017) datasets where the audio samples are affected by background music or noise. On the other hand, datasets like RAVDESS (Livingstone and Russo, 2018) and SAVEE (Haq and Jackson, 2010) provide clean audio and video samples but use categorical labelling.

Thus, we pre-train the face-channel of MCCNN combining Aff-Wild and AFEW-VA datasets with normalised arousal-valence labels ∈ [ − 1, 1] for each frame. The face-channel is trained with a 60 : 20: 20 (train, validation, test) data split reaching competitive Concordance Correlation Coefficient (CCC) scores of ***0.68*** for *arousal* and ***0.57*** for *valence* (compared to baselines provided by Zafeiriou et al. (2017) and Kossaifi et al. (2017)). The training details for the face-channel can be found in Supplementary Material. The face-channel is then used to classify facial images from the RAVDESS and SAVEE datasets, generating arousal and valence labels. These labels are then used to train the combined MCCNN network using audio-visual information. This approach is inspired from Lakomkin et al. (Lakomkin et al., 2017) who conclude that augmenting datasets using labels from one modality contributes positively towards improving the overall performance of the model. The MCCNN is trained for 200 epochs with the *Adam* optimiser converging to **CCC** scores of ***0.75*** for *arousal* and ***0.53*** for *valence*. The hyper-parameters for MCCNN are optimised using the Hyperopt^4^ python library. More details such as search-space for hyper-parameter tuning and the selected hyper-parameters (see Supplementary Material) as well as the training dynamics (see Supplementary Material) can be found in the Supplementary Material.

#### 4.1.2 Perception-GWR

For training the Perception-GWR model, feature vectors from the 200-d FC layer of the MCCNN are extracted. The GWR model is trained for 50 epochs with a maximum age of 50 for each neuron to allow for a neuron to be retained even if it fires only once per epoch. The habituation threshold (see Table 2 for details) controls the frequency of weight updates while the insertion threshold controls when a new neuron needs to be added. This results in a total of ***458*** **neurons** which sufficiently represent the entire training set ( ≈20 k data points). More details can be found in Supplementary Material. These neurons act as feature prototypes for the entire dataset, enabling a robust evaluation of the arousal-valence represented in the data samples. Figure 1 shows the Perception-GWR with each neuron plotted according to the arousal and valence it encodes by processing them using the MCCNN-classifier. The choice of the different thresholds is determined empirically, given the resultant GWR’s ability to represent the training set.

**TABLE 2:** Training parameters for the GWR Models.

Model	Habituation threshold	Insertion threshold	Max. Age	Context vectors	BMUs computed
Perception-GWR	0.2	0.5	50	–	2
Affective Memory	0.5	0.8	5	10	Mean Value
Time Perception Core	0.5	0.9	5	5	2
Social Conditioning Core	0.5	0.9	5	5	5
Robot Mood	0.5	0.9	5	10	Mean Value

#### 4.1.3 Affective Memory

The *affective memory* Gamma-GWR model consists of 10 context descriptors, implementing a temporal resolution of 10 time-steps. The model is trained following Eqs. 1–6. The chosen parameters (see Table 2) allow the model to map and remember the affective context for at least one complete interaction (5–8 s). Figure 1 shows the affective memory for the user with each neuron plotted according to the arousal and valence it encodes. The insertion and habituation thresholds control the update of existing neurons and add new neurons only when needed. A separate *affective memory* is created for each user interacting with the robot. More details can be found in Supplementary Material.

### 4.2 Mood Formation Under Affective Core Influences

To evaluate the impact of the different *affective core* influences on the mood formation of the robot, a separate test-set is generated consisting of 20 videos each from the KT Emotion Dataset (Barros and Wermter, 2017) and the OMG-Emotion Dataset (Barros et al., 2018) as both these datasets consist of clean audio-visual samples encoding different affective contexts. Each video is split into data-chunks representing 500 milliseconds of audio-visual information. The pre-trained Face Detector from the Dlib python library^5^ is used for extracting faces while the python speech features library^6^ is used to generate mel-spectrograms for each data-chunk. The data is input sequentially to the MCCNN and perception-GWR for feature extraction and representation providing inputs for the different Gamma-GWR networks for the *affective memory* and *affective core* biases.

The mood Gamma-GWR model is trained for 10 epochs taking as input, for every 500 milliseconds of audiovisual input, (i) 2 BMUs from the Perception-GWR encoded into the arousal-valence values they represent, (ii) the mean arousal-valence vector from the *affective memory*, (iii) five BMUs from the social conditioning Gamma-GWR and (iv) 2 BMUs from the time perception Gamma-GWR. Higher number of BMUs from the social conditioning Gamma-GWR increase the influence of social conditioning on the mood formation of the robot. Different combinations of *affective core* influences are explored to evaluate how these influence mood formation in the robot. A Two-Sided Mann-Whitney *U* test (Mann and Whitney, 1947) shows significant differences (see Table 3) in the resultant mood under different affective cores, compared to when no *affective core* (No Core) is used. The model is shown the same video sequences changing only the *affective core* between repetitions. Keeping all other variables constant, any change in the resultant mood can be attributed to the *affective core* used for computation. More details, with examples, can be found in Supplementary Material.

**TABLE 3:** Two-sided Mann-Whitney *U*-test results with the alternative hypothesis that the resultant mood under different affective cores is different from the *No Core* condition.

Affective core	Time perception	Social conditioning	Arousal		Valence	
			*U-statistic*	*p-value*	*U-statistic*	*p-value*
Patient	Patient	None	703.0	0.352	***559.0***	***0.020***
Impatient	Impatient	None	***186.0***	***<0.0001***	***211.0***	***<0.0001***
High-arousal	None	Excitatory	***317.0***	***<0.0001***	793.5	0.952
Low-arousal	None	Inhibitory	***500.5***	***0.003***	709.0	0.384
Patient High-arousal	Patient	Excitatory	***488.0***	***0.002***	766.5	0.748
Impatient High-arousal	Impatient	Excitatory	674.5	0.230	***251.0***	***<0.0001***
Patient Low-arousal	Patient	Inhibitory	***412.5***	***0.0002***	694.5	0.312
Impatient Low-arousal	Impatient	Inhibitory	***57.5***	***<0.0001***	***52.5***	***<0.0001***

Bold values signify statistical significance (*p* < 0.05).

**FIGURE 6:**
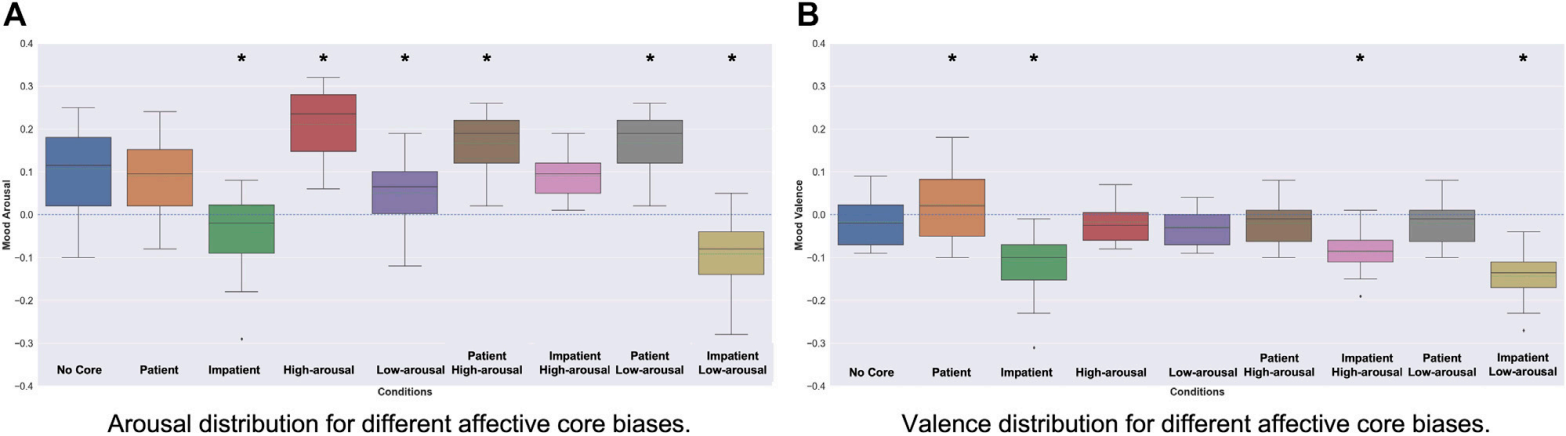
Arousal and Valence distributions for different Affective Core biases. All distributions are compared to the No Core condition to measure significant differences due to the Affective Core of the robot. A * denotes a significant variation compared to the No Core condition.

As can be seen in Figure 6A for arousal and Figure 6B for valence, the model, for the same input stimuli, results in different intrinsic mood for the robot based on the *affective core* influences used compared to the *No Core* condition which considers only the agent’s current perception and its *affective memory*. The arousal values show more deviation from the baseline due to the *excitatory* or *inhibitory* effect of the *affective core*. Since these biases predominantly affect the intensity of the robot’s intrinsic mood, the corresponding plots for the valence show much less deviation. On the other hand, the *patient* or *impatient* biases impact both the valence and arousal.

### 4.3 Pre-training Negotiating Behaviour for the Ultimatum Game

As discussed in Section 3.4.2, we propose to use the *mood* of the robot during the interactions as the basis to learn how to negotiate with the users, updating the robot’s offers based on the user’s responses. As the interaction with a particular user is limited and may not provide enough experience for the robot to learn an RL policy from scratch, we pre-train the negotiation model using synthetic data to emulate user behaviour during interactions.

We encode 20 video samples from the KT Emotion dataset (Barros and Wermter, 2017) using the multi-model affect perception model to generate arousal-valence encodings for each 500 ms of audio-visual data. This synthetic data is augmented by adding 500 randomly generated arousal-valence vectors, drawn from a standard normal distribution sliced to range ∈ [ − 1, 1], to cover the entire state-space. To match video dynamics, each added sample undergoes an interaction decay^7^ (forming a trajectory) emulating affective responses from a respondent that witnesses consecutive unfair offers from the robot and rejects them. Furthermore, the acceptance of the offers made by the robot is modelled in a stochastic manner (see Eq. 7) based on the fraction of the resources being offered to the participant.

$$p\left(\text{acceptance}\right)=\left\{\begin{array}{cc} 1,\; & \text{if }\;o\mathrm{ff}er\geq \;0.7\\ o\mathrm{ff}er,\; & \text{if }0.7\;>o\mathrm{ff}er\geq \;0.5\\ 0.1,\; & \text{if }0.5\;>o\mathrm{ff}er\geq \;0.4\\ 0,\; & \text{otherwise} \end{array}\right.$$ (7)

For effectively training the DDPG-based RL model to negotiate with the users we modelled the reward function with two objectives; (i) trying to *win* the negotiation by keeping a higher share for the robot and (ii) maintain a positive intrinsic *mood* for the robot. The two components of the reward are explained as follows:

• **Offer Reward:** The robot is given intermediate positive rewards if the new offer increases the respondent’s share (*h*) while keeping its share (*r*) >=50% . These rewards smoothen the learning curve, guiding the robot to an optimal behaviour. The robot, cumulatively, receives positive rewards if it increases the respondent’s share but starts getting penalised if it reduces its own share below 50*%*.
• **Mood Reward:** The robot computes a (cosine) distance measure between its previous and new mood state and receives a positive reward for a positive change in its mood. As the robot’s mood reflects its appraisal of the respondent’s affective state, the robot should learn to evoke positive responses to its offer. An alternate to this could be to directly use the user’s affective state to compute the *mood reward* but this does not take into account intrinsic influences such as affective memory or the affective core of the robot which may modulate its perception of the user.

**FIGURE 7:**
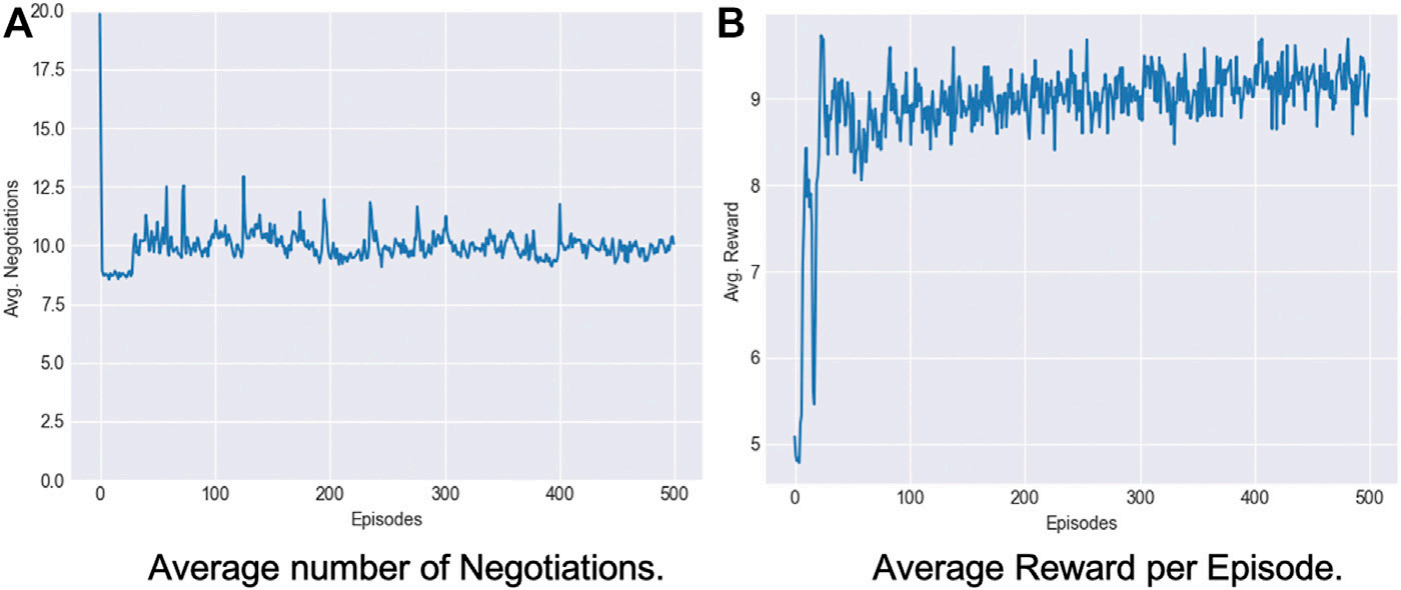
Robot learning to negotiate, converging an offer >45% of the resources in ≈10 interactions.

The net reward for the robot for each negotiation round is the summation of the *offer reward* and the *mood reward* balancing the two goals of improving its offers to the respondent while keeping a higher share for itself. The model learns to balance both the offer and mood reward and converges to offering 40–60*%* points, yielding an optimal reward for the robot (see Figure 7B). The average number of interactions reduce to ≈10 as the model learns to find an optimal offer that faces fewer rejections (see Figure 7A).

## 5 User Study: Negotiating With Participants

Motivated by the results presented in Table 3, where the choice of affective core influences results in a significant difference in the robot’s mood, we investigate whether this difference can be translated into different negotiation strategies in the Ultimatum Game scenario (see Section 3.4.1). We conduct a user study assessing how different participants evaluate the NICO robot’s behaviour during negotiations under specific affective core influences. In addition to the participants’ subjective experience interacting with the robot, we quantitatively measure objective performance metrics like success-rate (acceptance of the robot’s offer), mean accepted offer value, and the average number of interactions needed to negotiate a split of resources.

The user study was conducted with 31 (*n*) participants (20 male, 11 female) from 16 countries in the age-group of 18–49, with a majority (*n*′ = 24) of the participants in the age group of 25–34. All participants, recruited amongst university students and employees, reported conversational proficiency in English (the language used to model interactions). The participants were briefed about the objectives of the experiment and the interaction procedure and they provided *informed consent* for their participation. The consent form and the experiment protocol were approved by the Ethics Commission^8^ of the Department of Informatics, University of Hamburg.

The experiment set-up (see Figure 4) consists of an artificially well-lit room to exclude effects of changing natural lighting conditions. The participants and NICO are positioned across a round-table, opposite to each other with the *Bonbons* placed on the table along with a microphone.

### 5.1 Experiment Conditions

The user study is conducted as a *between-group* study with two condition groups. Each group consists of two sub-conditions implementing the *No Core* condition as the *baseline*, along with one of the *measured* conditions. In the *No Core* condition, the robot is not embedded with any *affective core* and considers only the perception input for its intrinsic mood. For each participant, the two condition groups are:

• **Patient High-arousal**: In this group, the *measured* condition involves the robot with *patient* time perception and *excitatory* social conditioning biases to influence its mood formation. A total of 16 participants (10 male and six female) were randomly assigned to this condition group.
• **Impatient Low-arousal**: In this group, the *measured* condition involves the robot embedded with an *impatient* time perception and *inhibitory* social conditioning bias that influence mood formation. The second condition group consisted of 15 randomly assigned participants (10 male and five female).

Each participant in either of the condition groups witnessed both the sub-conditions, that is, the *No Core* condition and one of the *measured* conditions, one after the other. Within a particular condition group, the order in which the two sub-conditions were shown was randomised to reduce the effect of any bias arising from the ordering of these conditions. The choice of Patient High-arousal and Impatient Low-arousal conditions is motivated by the results presented in Table 3 where a significant difference in the resultant robot mood is witnessed compared to the *No Core* condition. Even though there are significant results witnessed for other conditions as well, these two conditions modelled both time-perception and social conditioning biases in opposite directions thus, serving as a good basis for comparing mood formation in the robot. The *No Core* condition acts as the anchor against which the other two conditions are measured.

### 5.2 Experiment Protocol

Once the participants are assigned to a condition group, they are introduced to the experiment set-up where NICO greets them by modelling a short interaction with the participants informing them about the rules of the game. Google Text-To-Speech python library^9^ (ver. 1.1.8) is used to generate NICO’s voice. During this interaction, NICO asks the participants about their excitement towards participating in the experiment. With this data, it builds a model of its *affective memory* and intrinsic *mood* as a starting point for both sub-conditions. After the introduction round, NICO starts negotiating with the participant, randomly loading the first sub-condition. The negotiations progress in two distinct phases:

• **Offer phase:** NICO makes an offer to the participants which they can accept (saying ‘Yes’) or reject (saying ‘No’). If the offer is accepted, the interaction culminates while a rejection results in NICO asking the participant to explain their rejection while monitoring their affective responses as they describe their opinion about the offer.
• **Update phase:** Observing the participants’ responses, NICO models its *mood*, as a response. The mood represents the current state of the robot and is used to compute a new offer for the participants using the RL model (see Section 4.3).

Although the sub-game perfect Nash equilibrium prediction for the Ultimatum Game is achieved when the proposer offers an infinitesimal (smallest non-zero value; in this case 1) share to the respondent, which they accept, many studies have shown that this is not in agreement with experimental observations (Oosterbeek et al., 2004; Schuster, 2017) and accepted offers may vary around 40%. To encourage negotiations (at least once) between the robot and the participants, we enforce the first offer made by the robot to be a random unfair offer between 1–20 points. Negotiations continue until the participant either accepts the offer or rejects 20 consecutive offers (empirically defined limit). The participants are told that the robot shall abort the negotiation if a *stalemate* is reached, blinding them from this limit to avoid any behavioural conditioning. No significant difference (*H* = 0.85, *p* = 0.65) is witnessed using the Kruskal-Wallis H-test (Kruskal and Wallis, 1952) in the *First Offers* made by the robot between the conditions and thus, has no measurable effect on the number of interactions between the conditions.

After each sub-condition, the participants fill out a *pseudonymised* 3-part questionnaire about their experience with the robot. Finally, participants are debriefed and informed about the condition group they were assigned. In the absence of any monetary compensation, as a reward for their participation, the participants are offered all the bonbons.

### 5.3 Objective Evaluations

To evaluate the robot’s performance under different conditions, we measure several objective metrics (see Table 4). The *success rate* denotes the fraction of participants that accepted the robot’s offer. The *number of interactions* denotes the number of rejections, on average, before an offer was accepted while the *average accepted offer* represents the average offer value accepted by the participants. In the case, no split could be negotiated, we examine the final offer before the negotiation was aborted by the robot. Table 4 also reports the fraction of offers where the participants were offered 50*%* or more of the points by the robot.

**TABLE 4:** Quantitative Analysis of Objective Measures on NICO’s performance in the Ultimatum Game under different experimental conditions.

Measured value	Baseline (N = 31)	Patient high-arousal (N = 16)	Impatient low-arousal (N = 15)
Number of Interactions	08.71 ± 0.64	09.35 ± 1.13	08.33 ± 1.20
First Offer	15.00 ± 0.90	14.00 ± 1.20	15.00 ± 1.00
Accepted Offer	44.00 ± 2.00	45.00 ± 1.60	43.00 ± 2.00
Final Offer (If rejected)	49.00 ± 0.30	47.00 ± 0.90	50.00 ± 0.30
When Offered >=50 %	77*%*	62*%*	80*%*
Success Rate (Offer Accepted)	90*%*	87*%*	80*%*

As each measured condition is evaluated with respect to the *baseline* (No Core) condition, the two measured conditions cannot be compared to each other directly, as such. This is possible only if the respective *baseline* measurements in the two groups do not vary significantly. A Two-sided Mann-Whitney *U* test (Mann and Whitney, 1947) shows no significant difference (*p* > 0.05) across any dimension between the two baselines, either in the above-mentioned objective metrics or the user evaluations across the different questionnaires (see Section 5.4). This allows for the two *measured* sub-conditions to be compared to each other, directly.

The *Patient High-arousal* condition, on average, took longer (9.35 ± 1.13) than the *baseline* condition (8.71 ± 0.64) to get the participant to accept an offer with a large effect size (*G* = 0.77) shown using the Hedges’ G test measuring the effect of the condition on the different metrics. The *Impatient Low-arousal* condition however, needed fewer interactions (8.33 ± 1.20) than the baseline condition with a medium effect size (*G* = 0.44) in the other direction. Comparing the two measured conditions directly thus, shows a large effect size (*G* = 0.87) for the number of interactions where the robot under the *Patient High-arousal* condition negotiated for longer than under the *Impatient Low-arousal* condition. Although pair-wise comparisons did not show significant differences (*p* > 0.05), the above-mentioned Hedges’ G values show a medium-to-large effect size of the condition on the length of the negotiations. Furthermore, under the *Impatient Low-arousal* condition, the robot was able to reach an offer >=50% of the points for 80*%* of the participants as compared to 62*%* for the *Patient High-arousal* condition, further indicating how the condition assigned impacted the robot’s offers. Despite reaching a higher offer more often, the success rate (80*%*) and the mean accepted offer (43.00 ± 2.00) for the robot in the *Impatient Low-arousal* condition was lower than the success rate (87*%*) and accepted offer value (45.00 ± 1.60) for the *Patient High-arousal* condition. Yet, this difference was not significant (*p* > 0.05). As participants increasingly received more points in the *Impatient Low-arousal* condition, they exhausted the 20 offers, anticipating the robot to increase the offer further. This observation is validated by the *Final Offer* value, that is, the offer before aborting the interaction, being higher for the *Impatient Low-arousal* condition (50.00 ± 0.30) as compared to the *Patient High-arousal* condition (47.00 ± 0.90) with a large effect size (*G* > 2.0) between the two measured conditions.

### 5.4 Subjective Evaluations

Since the participants’ subjective evaluation of the robot’s negotiation strategy influences their acceptance or rejection, objective factors provide only partial information about the robot’s overall performance. Thus, participants’ evaluations on the 3-part Likert-scale questionnaire, based on the GODSPEED (Bartneck et al., 2008), Mind Perception (Gray et al., 2007) and Asch’s Personality Impression tests (Asch, 1946), are examined.

#### 5.4.1 GODSPEED

The GODSPEED test (Bartneck et al., 2008) is used to measure participants’ impression of the robot on anthropomorphism, animacy, likeability, perceived intelligence and perceived safety. A one-sided Mann-Whitney *U* test is conducted for all dimensions with an alternative hypothesis that the *Impatient Low-arousal* condition is rated higher than *Patient High-arousal*. The results show no significant differences (*p* > 0.05) in any dimension despite some evidence for the robot rated as more *natural* (*U* = 154.5, *p* = 0.07), *human-like* (*U* = 158.0, *p* = 0.053) and *conscious* (*U* = 158.0, *p* = 0.061) under the *Impatient Low-arousal* condition. More details on the GODSPEED evaluations can be found in Supplementary Material.

#### 5.4.2 Mind Perception

The Mind Perception test (Gray et al., 2007) measures *agency* and *experience* for attributing a *mind* in an entity (in this case, NICO). The robot is evaluated on its ability to experience *fear*, exercise *self-control*, feel *pleasure*, *remember* the participant, feel *hunger* and to *act morally*. Based on these factors, the robot’s *agency* and *experience* under different conditions is concluded. A one-sided Mann-Whitney *U* test is conducted with the alternative hypothesis that the *Impatient Low-arousal* condition is rated higher on *agency* and *experience*. No significant difference (*p* > 0.05) can be concluded between the two conditions across any dimension. More details on the Mind Perception evaluations can be found in Supplementary Material.

#### 5.4.3 Asch’s Formation of Impressions of Personality

**FIGURE 8:**
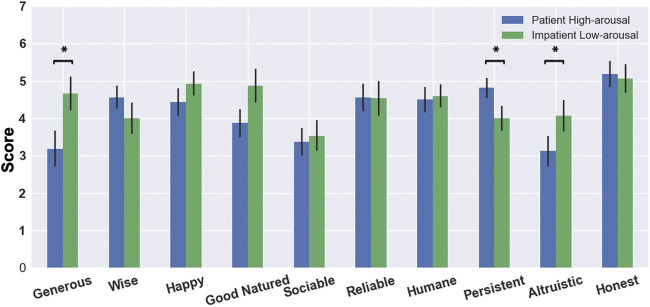
Asch’s Test results with mean and 95*%* CI for individual dimensions comparing the two measured conditions. A * denotes a significant difference between the two conditions.

Asch’s study (Asch, 1946) measures the impact of independent behavioural traits on the overall impression of an individual. Here, participants evaluate NICO on 10 different parameters. Their impressions for the robot under the two measured conditions can be seen in Figure 8. For all dimensions, except *wisdom* and *persistence*, the *Impatient Low-arousal* condition is rated higher, while in these dimensions, the *Patient High-arousal* condition is rated higher. A one-sided Mann-Whitney *U* test conducted on all dimensions shows significant results (*p* < 0.05) for the *generous* (*U* = 67.0, *p* = 0.018) and *altruistic* (*U* = 74.0, *p* = 0.033) dimensions in favour of the *Impatient Low-arousal* condition (see Table. 5). Post-hoc analyses reversing the order of effect shows a significant difference between the measured conditions for the *persistence* (*U* = 75.0, *p* = 0.034) dimension (*highlighted* in Table 5) as well, albeit in favour of the *Patient High-arousal* condition. These results underline the effect of the condition on the participants’ perception of the robot. The robot under the *Patient High-arousal* condition negotiates for longer (see Section 5.3) and thus is witnessed as more *persistent* as it tries to keep a higher share for itself. On the other hand, under the *Impatient Low-arousal* condition, it offers higher increments on successive offers, giving away more points easily. As a result, it is rated to be more *altruistic* and *generous* in its negotiation. Despite some evidence supporting the alternative hypothesis for the *good-natured* dimension (*U* = 79.0, *p* = 0.052), no other significant difference is witnessed between the conditions.

**TABLE 5:** One-sided Mann-Whitney *U* test for Asch’s Test with alternative hypothesis that the Impatient Low-arousal condition is rated higher.

Dimension	U-statistic	*p*-value	Dimension	U-statistic	*p*-value
**Generous**	**67.0**	**0.018**	Reliable	120.5	0.516
Wise	142.5	0.825	Humane	119.0	0.492
Happy	95.0	0.161	*Persistent*	*165.0*	*0.969*
*Good Natured*	*79.0*	*0.052*	**Altruistic**	**74.0**	**0.033**
Sociable	112.5	0.387	Honest	123.5	0.564

Bold values signify statistical significance (*p* < 0.05).

## 6 Discussion

In this work, we propose a novel framework that explores a robot’s appraisal of its interactions with individuals to ground evolving affective representations that not only consider the behaviour of the participant during an interaction (see Section 3.1), but also understand its impact on the conversation (see Section 3.3), learning how to respond to them (see Section 3.4). This is guided by the affective and behavioural disposition of the robot (see Section 3.2) which has a significant impact on its affective appraisal.

Quantifying the affective impact of the duration of an interaction is beneficial for a robot, particularly in collaborative HRI scenarios. A *patient* time perception can be helpful in dealing with *negative* situations as it will allow the robot to maintain a positive outlook during the interaction. This can be beneficial for robots acting as companions for humans in different collaborative scenarios such as being caretakers for the elderly and tutors for the young. Conversely, *impatience* may result in a significantly lower intrinsic state of the robot, rapidly decaying its mood as the interaction progresses. This may enhance spontaneity in robot behaviour as it finds ways to resolve a negotiation quickly, to avoid negative intrinsic states. In our experiments, we see evidence for this as the robot average number of interactions under the *Patient High-arousal* condition is higher than in the *Impatient Low-arousal* condition (see Table 4). High arousal interactions can cause the robot to form *excitatory* (or high-arousal) tendencies that amplify its affective state. While interacting with the users, the robot is easily excitable, experiencing every situation in the *extreme*. An *inhibitory* (or low-arousal) conditioning, on the other hand, results in a *subjugated* behaviour of the robot, diminishing the impact of affective interactions and adopting an inert approach towards its interaction with the users. Combining time perception and social conditioning allows for modelling specific affective and behavioural dispositions in the robot with the two influences either complementing each other, for example, *Patient high-arousal* and *Impatient low-arousal*, or contrasting each other, for example, *Patient low-arousal* and *Impatient high-arousal* conditions. These conditions have a distinct impact on the affective appraisal of the robot (see Table 3) as the resultant mood does not merely mimic the user’s affective state but reflects the robot’s intrinsic dispositions.

The robot’s intrinsic mood, modulated by specific *affective core* dispositions, as well as history with a user, governs its negotiations with the user. This allows the robot to share a common view of the interaction with the user and yet, have a distinct response towards it rather than merely mimicking the users’ behaviour. In our experiments, the *patient high-arousal* robot is witnessed to be more persistent, driving a harder bargain with users while the *impatient low-arousal* robot, on the other hand, is more giving and generously offers more points. This is highlighted in the objective metrics (see Section 5.3) evaluating the robot’s performance as well as the subjective evaluations by the participants (see Section 5.4).

During interactions, based on the robot’s behaviour, the participants were witnessed adopting different negotiating strategies. While some approached the interaction donning a more *commanding* role, strongly arguing with the robot to yield, others followed a fawning approach trying to manipulate the robot by smiling more often and *requesting* more points. Both strategies, given the experiment condition and the expressiveness of the participants, worked to some extent with the robot offering, more points to the user (as high as 52*%*) in some cases. Furthermore, at the beginning of the interactions, some participants were more *conscious* and *distant*, but as the interaction progressed, they became more *open* and *proactive* in the interaction. This is seen in the reasoning provided by them for their rejection which ranged from a cold and direct “*I want more points*” later to a more expressive and layered *“Come on, NICO. This isn’t fair. You can do better”*. This suggests that as the interaction progressed, the robot was able to *engage* the users by understanding and sharing their view of the negotiation. It exhibited responsiveness towards the users’ negotiating strategies, initially yielding to their demands for more points but, as the interaction progressed, it adapted its negotiation strategy, encouraging the users to also adapt.

Learning appropriate negotiating behaviour for the robot was premised upon two factors; affective responses of the participants towards the robot and the factoring in rejected offers to learn to make acceptable updates on the offers. The reward function design for the RL model assigned equal weights to both these objectives such that the robot tried to offer a “fair” split of the resources keeping a sizeable share for itself while, at the same time, learning an update strategy that will evoke positive responses from the participants. Future works and extensions will focus on dynamically learning to prioritise these two objectives while also establishing their individual contributions towards learning acceptable negotiating behaviours in social robots.

To simplify the HRI evaluation, in this work we compared only two *affective core* biases; Patient High-arousal and Impatient Low-arousal, where the effect of time perception and emotional actuation complemented each other. Although we investigate how each individual core influence impacts mood formation in the robot, it will be interesting to also evaluate if and how these influences are translated to the negotiation behaviour adopted by the robot. This will also help illuminate individual contributions of these underlying influences on realising appropriate negotiation behaviour for the robot.

Despite the participants noticing significant differences in its negotiating strategy (see Table 5), the general perception of the robot did not change under different conditions. This could be due to the fact that the only difference between conditions is in how the robot updates its offers. The interaction structure, what is said and robot’s facial expressions remain the same between conditions. This difference is perhaps *too subtle* to induce an overall change in perception towards the robot. In future, we would also like to modulate the dialogues and the robot’s facial expressions to reflect the robot’s mood as well as adding phrases that reflect the *affective core* condition.

## 7 Conclusion

In this work, we present a comprehensive framework for modelling affect-driven modulations on robot behaviour in collaborative HRI scenarios. Using a multi-modal affective appraisal model, it forms an evolving understanding of human behaviour, yielding intrinsic responses in the robot towards the user that constitute its own affective state. This intrinsic state is used to learn negotiating behaviour in the Ultimatum Game. The *affective core* of the robot realises specific behavioural dispositions in the robot that influence its intrinsic state as well as its behaviour. This is beneficial for the robot to dynamically interact with users rather than following static, pre-determined behaviour policies.

The results from the user study show that the participants were able to notice the effect of the *affective core* on factors such as *generosity* and *persistence* which directly evaluated the robot’s behaviour in the Ultimatum Game. The general impression of the robot, however, did not change significantly. Further experimentation is needed, involving longitudinal studies with more participants, to conclude any significant impact on the overall impression of the robot. Furthermore, in the user study, the *affective core* models are pre-trained and used after freezing the weights for the Gamma-GWR model. This was done to simplify the training and eliminate the effect of changing *affective core* biases on the performance of the robot. It will be interesting to let these models to grow and adapt as the robot interacts with more users, allowing the robot to change its outlook on the users as it interacts with them.

## Data Availability

Publicly available datasets were analyzed in this study. This data can be found here: https://ibug.doc.ic.ac.uk/resources/afew-va-database/, https://ibug.doc.ic.ac.uk/resources/aff-wild2/, http://kahlan.eps.surrey.ac.uk/savee/Download.html, https://zenodo.org/record/1188976#.YLOGAi9Q3DU, https://github.com/knowledgetechnologyuhh/OMGEmotionChallenge, https://www2alt.informatik.uni-hamburg.de/wtm/publications/2017/BW17/Barros-Affective_Memory_2017-Webpage.pdf.

## Footnotes

1
http://python-speech-features.readthedocs.io.

2
https://github.com/hyperopt/hyperopt.

3 (*r*, *h*)-vector where *r* is the robot’s share and *h* is the participant’s share.

4
https://github.com/hyperopt/hyperopt.

5
http://dlib.net.

6
http://python-speech-features.readthedocs.io.

7 ({*a*, *v*} = exp(−*τt*); *τ* = 0.02).

8
https://www.inf.uni-hamburg.de/en/home/ethics.html.

9
https://pypi.python.org/pypi/gTTS.
