# Supplementary material for: Affect-Driven Learning of Robot Behaviour for Collaborative Human-Robot Interactions
Source: Front Robot AI. 2022 Feb 21;9:717193. doi: 10.3389/frobt.2022.717193 (PMC8898942; doi:10.3389/frobt.2022.717193)
Supplement: Supplementary file 1 [file DataSheet1.pdf]

Supplementary Material

S.1 TRAINING DETAILS AND HYPER-PARAMETERS

S.1.1 Hyper-parameter Search for the MCCNN

The hyper-parameters for the Multi-Channel Convolutional Neural Network (MCCNN) are tuned by conducting a grid-search over different parameter settings by optimising the model for arousal-valence prediction. The search space for the hyper-parameter tuning can be seen in Table S1.

Table S1. Search space used for hyper-parameter optimisation for the MCCNN model. $\mathcal{U}$ denotes a uniform distribution sampled between the given minimum and maximum value.

Parameter	Value	Parameter	Value
Batch Size	$choice(20, 50, 64, 128)$	MCCNN FC Layer Units	$choice(128, 200, 256, 512)$
Learning Rate	$\mathcal{U}(0.001, 0.0001)$	Optimizer	$choice(adam, adamax, rmsprop)$
**Vision Channel**		**Speech Channel**	
Layer1: Filter Maps	$choice(8, 16, 32)$	Layer1: Filter Maps	$choice(8, 16, 32)$
Layer1: Filter Size	$choice(5, 7, 9)$	Layer1: Filter Size	$choice(5, 7, 9, 10)$
Layer2: Filter Maps	$choice(8, 16, 32)$	Layer2: Filter Maps	$choice(8, 16, 32)$
Layer2: Filter Size	$choice(5, 7, 9)$	Layer2: Filter Size	$choice(5, 7, 9, 10)$
FC Layer Units	$choice(128, 256, 512)$	FC Layer Units	$choice(128, 256, 512)$
Dropout-Rate	$\mathcal{U}(0, 1)$	Dropout-Rate	$\mathcal{U}(0, 1)$

Each parameter permutation was run 10 times to account for the random seed and best performing parameter settings were selected. The selected hyper-parameters for the MCCNN model can be seen in Table S2.

Table S2. Selected Hyper-Parameters for the MCCNN Network, optimised using the Hyperopt python Library.

Vision Channel		Audio Channel		MCCNN	
**Layer 1**		**Layer 1**		Epochs	200
Filter Maps	32	Filter Maps	32	Batch Size	64
Filter Size	(9×9)	Filter Size	(9×10)	Optimiser	Adam
Regularisation	L1-Norm	Regularisation	L1-Norm	Learning Rate	0.001
Dropout-Rate	0.5	Dropout-Rate	0.5	Weight Initialiser	Glorot Normal
Max-Pooling	(2×2)	Max-Pooling	(2×2)	FC Layer Units	200
**Layer 2**		**Layer 2**		Activation	ReLU
Filter Maps	32	Filter Maps	32		
Filter Size	(7×7)	Filter Size	(7×7)		
Regularisation	L2-Norm	Regularisation	L2-Norm		
Dropout-Rate	0.5	Dropout-Rate	0.5		
Max-Pooling	(2×2)	Max-Pooling	(2×2)		
**FC Layer**		**FC Layer**			
Units	512	Units	512		

S.1.2 Training the Face-Channel

The Face-channel of MCCNN is pre-trained on the Aff-Wild and AFEW-VA datasets with normalised arousal-valence labels $\in [-1, 1]$ for each frame. The training/validation loss dynamics and model performance in terms of the Concordance Correlation Coefficient (CCC) scoresis shown in Figure S1. As can be seen, the model achieves competitive validation CCC scores of $\mathbf{0.68}$ for *arousal* and $\mathbf{0.57}$ for *valence*.

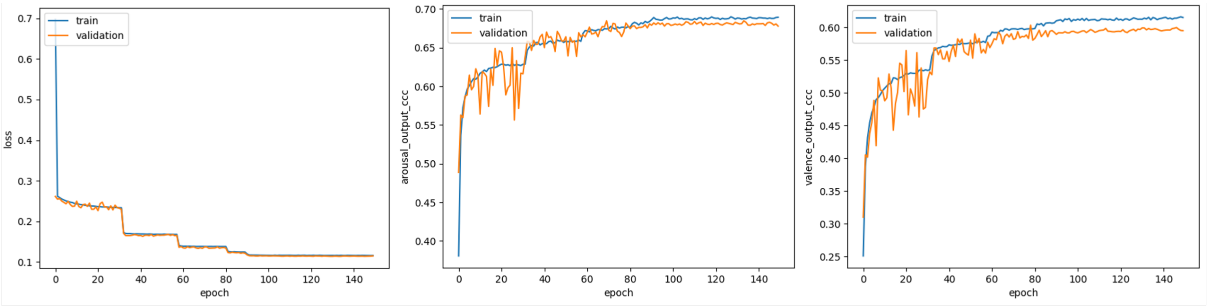

(a) Training/Validation Loss. (b) Arousal CCC. (c) Valence CCC.

Figure S1. Training and Validation Loss, and Model Performance in terms of Arousal and Valence CCC scores for Face Channel Pre-training.

S.1.3 Training the MCCNN

The Face-channel is used to label the SAVEE and RAVDESS datasets in terms of the arousal-valence values ($\in [-1, 1]$) they represent. The resultant training and validation distributions can be seen in Figure S2.

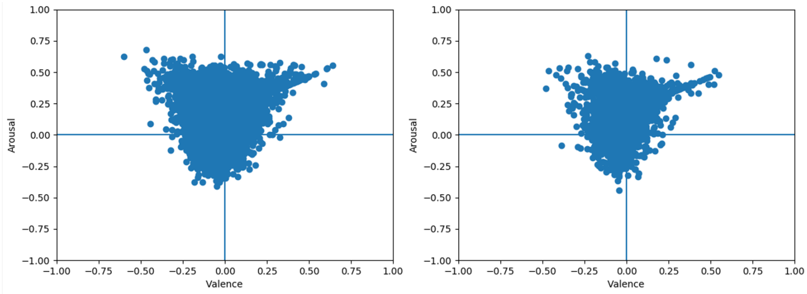

(a) Training Set Distribution. (b) Validation Set Distribution

Figure S2. Training and Validation Data Distribution for Training the MCCNN.

The MCCNN is then trained on this data for 200 epochs with the *Adam* optimiser converging to **CCC** scores of $\mathbf{0.75}$ for *arousal* and $\mathbf{0.53}$ for *valence*. The training/validation loss dynamics and model performance in terms of CCC scores can be seen in Figure S3.

S.1.4 Training the Perception-GWR

The trained MCCNN network acts as a multi-modal feature extractor. The extracted training-set features are then used to pre-train the Perception-Growing-When-Required (GWR) model in an unsupervised manner. The Perception-GWR learns robust feature prototypes to adequately represent the training set, mitigating the effect of spurious outliers. The PCA-plot for the learnt feature representations, encoded into

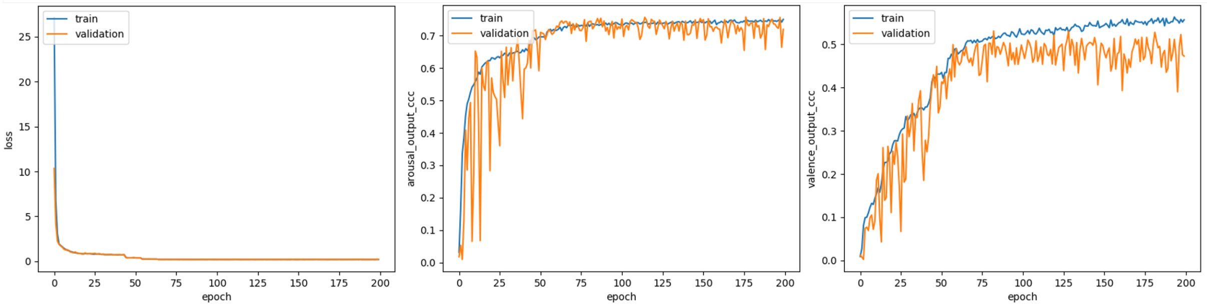

(a) Training/Validation Loss. (b) Arousal CCC. (c) Valence CCC.

Figure S3. Training and Validation Loss, and Model Performance in terms of Arousal and Valence CCC scores for MCCNN Training.

the arousal-valence values each neuron represents, can be seen in Figure S4 that learns feature prototypes for the training data distribution seen in Figure S2 (a).

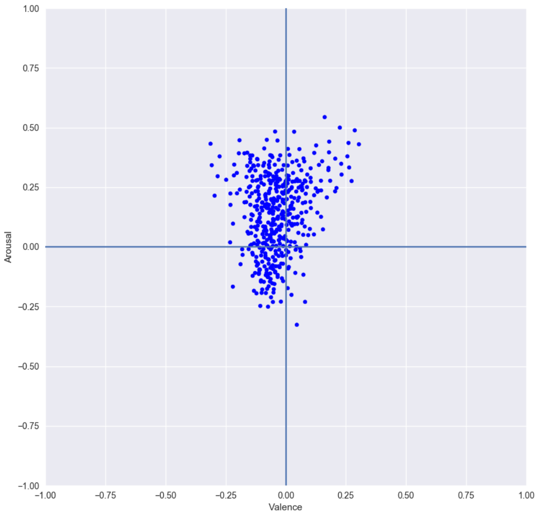

Figure S4. The Perception-GWR encoding feature representations extracted from the training data using the MCCNN.

S.1.5 Training Affective Memory Gamma-GWR

For each user interacting with the robot, it must create a separate affective memory representing past interactions with the user. BMUs from the Perception-GWR are used to train the Mood Gamma-GWR, setting the Gamma-filtering (temporal context) $K = 10$. An example of the Affective Memory Gamma-GWR training on a video sample from the KT-Emotion dataset can be seen in Figure S5.

S.1.6 Training Mood Gamma-GWR

The Mood of the robot represents its intrinsic affective response towards the user based on its perception of the user as well as intrinsic factors such as its affective memory as well as its inherent affective core dispositions. As can be seen in Figure S6, given the inherent affective core of the robot, the same affective stimuli can be experienced differently, resulting in a significantly different *mood* for the robot.

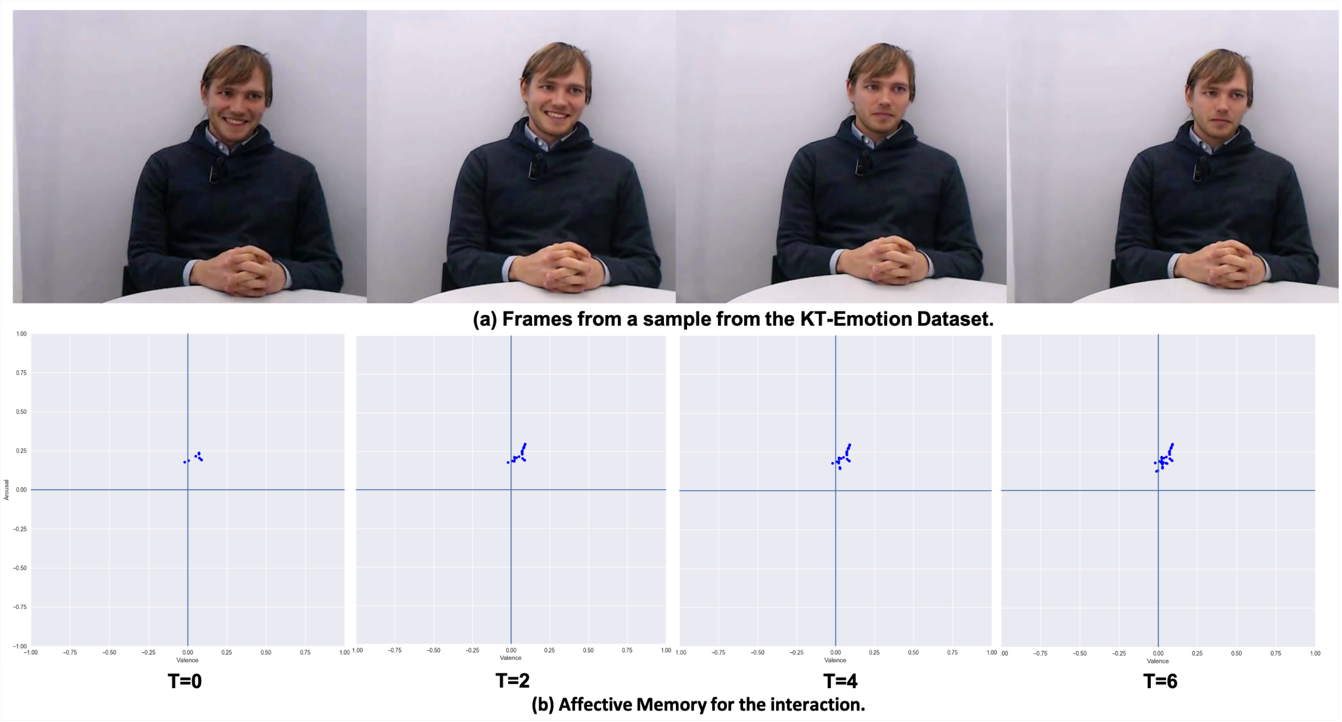

Figure S5. The Affective Memory Gamma-GWR grows as the agent interacts with the user over time.

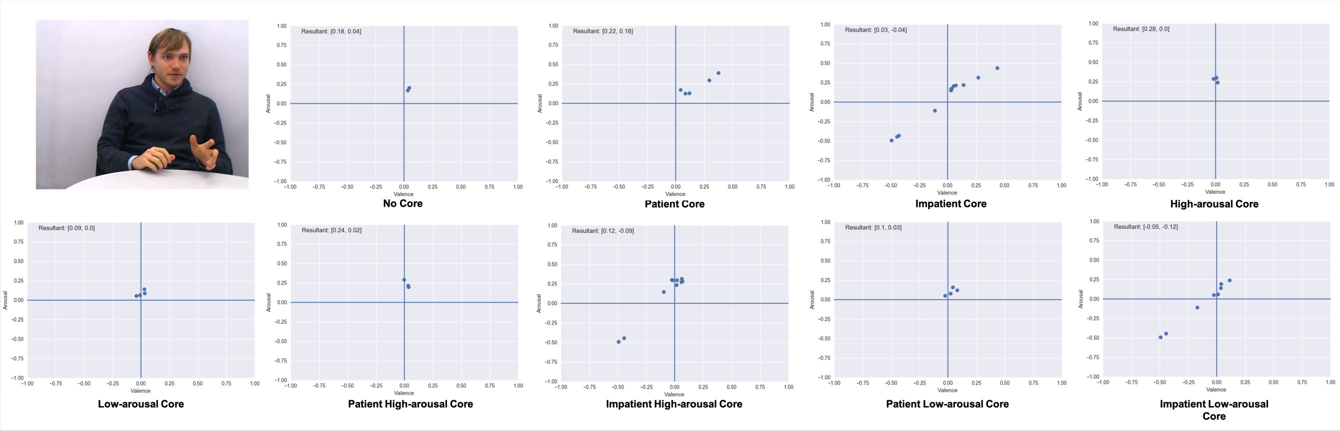

Figure S6. Robot's Mood under different Affective Core dispositions for the same affective input.

S.1.7 Learning Negotiating Behaviour

At any given time during the interaction, the robot observes the facial expressions as well as the speech of the participants to evaluate their affective response towards the robot's offer. The facial expressions and speech signals are processed using the MCCNN architecture (see Section 3.1.1 of the manuscript) and the extracted multi-modal feature vectors are used to encode feature prototypes using the Perception-GWR model. These feature prototypes are then encoded into arousal-valence values (see Section 3.1.2 of the manuscript) to represent the current perception of the robot. This current perception, modulated by different intrinsic and extrinsic factors influences the robot's mood, which is again summarised in terms of arousal and valence values (see Section 3.3 of the manuscript). These arousal-valence values and the previously

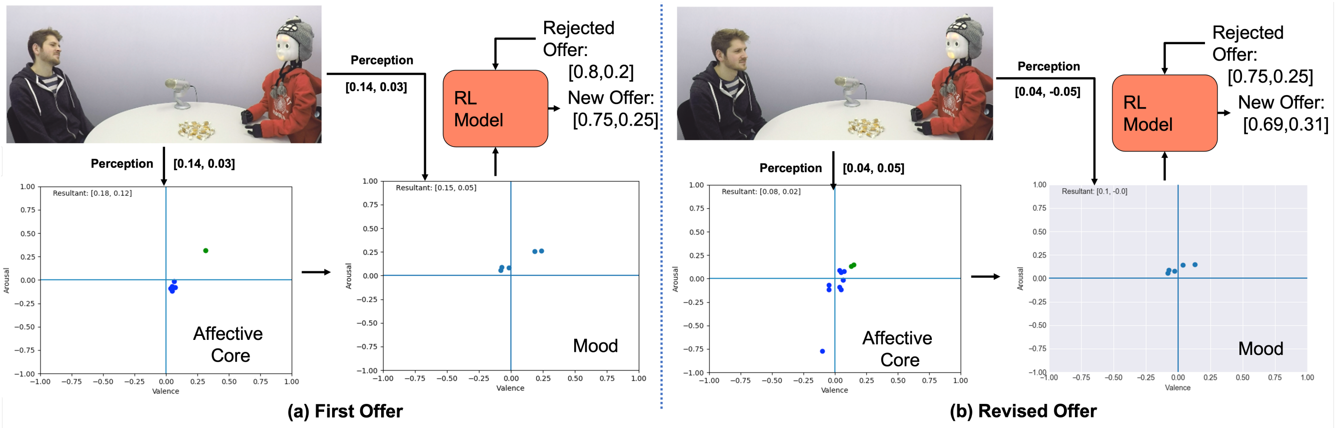

Figure S7. Robot Learning to Revise its offer based on the participants' affective behaviour during the interactions, modulated by its affective core.

rejected offer are used to generate an update on the previous offer, resulting in a new offer made to the participant (see Section 3.4.2 of the manuscript). Figure S7 illustrates how the robot updates its offer to the participant, based on the rejected offer as well as their affective behaviour towards the robot.

S.2 ADDITIONAL RESULTS FROM THE USER STUDY

S.2.1 GODSPEED Results

The GODSPEED test is used to measure a participants' impressions of the robot on criteria like anthropomorphism, animacy, likeability, perceived intelligence and perceived safety. Each dimension of the questionnaire can be independently evaluated to get a deeper understanding of the participants' impressions of the robot. The means and the 95% confidence intervals for each of the dimensions comparing the two measured conditions can be found in Figure S8. The results of the one-sided Mann-Whitney U test conducted on individual dimensions can be seen in Table S3.

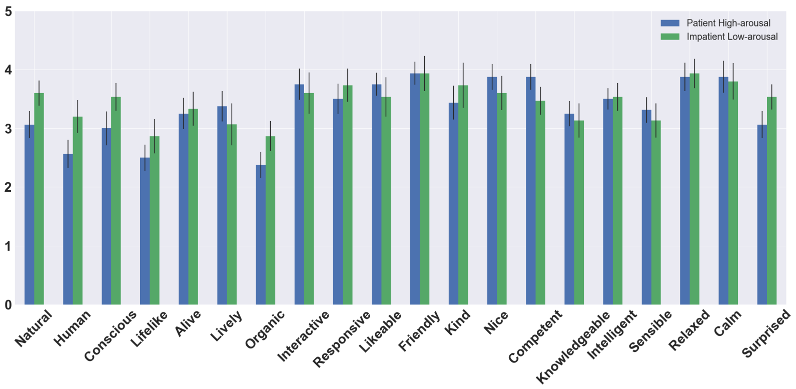

Figure S8. GODSPEED test results with means and 95% confidence intervals for individual dimensions comparing the Patient High-arousal condition with the Impatient Low-arousal condition.

S.2.2 Mind-Perception Results

Based on the participants evaluating the robot on factors such as experiencing *fear*, exercising *self-control*, feeling *pleasure*, *remembering* the participant, feeling *hunger* and *acting morally*, the agency

Table S3. Results of the one-sided Mann-Whitney U test evaluating the GODSPEED test results with the alternative hypothesis that the Impatient low-arousal condition was rated higher.

Dimension	U Statistic	p-value	Dimension	U Statistic	p-value
Natural	154.5	0.076	Friendly	129.0	0.362
Human	158.0	0.053	Kind	144.5	0.163
Conscious	158.0	0.061	Nice	106.5	0.721
Lifelike	142.0	0.181	Competent	92.5	0.882
Alive	124.0	0.442	Knowledgeable	112.0	0.638
Lively	103.0	0.762	Intelligent	123.5	0.449
Organic	155.0	0.075	Sensible	107.5	0.705
Interactive	116.5	0.565	Relaxed	124.5	0.434
Responsive	138.0	0.236	Calm	117.0	0.557
Likeable	115.0	0.591	Surprised	153.0	0.081

and experience exhibited by the robot under different conditions are concluded. The mind-perception evaluation for the robot under the Patient High-arousal and the Impatient low- arousal conditions can be seen in Figure S9. No significant difference can be concluded between the two conditions for either *agency* of *experience* dimensions, as can be seen in Table S4.

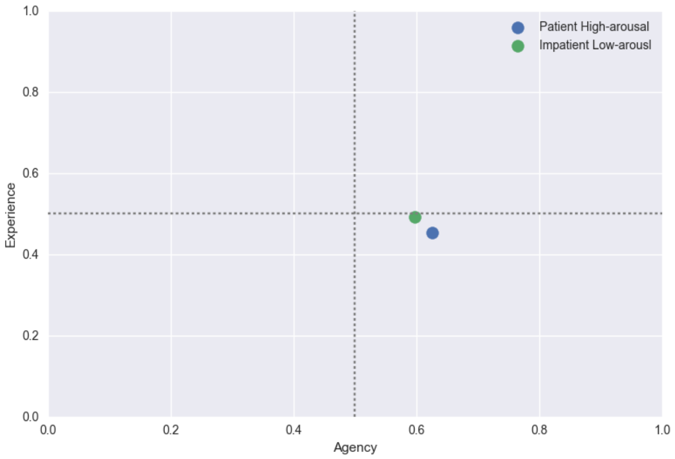

Figure S9. Mind Perception results comparing the Patient High-arousal and Impatient Low-arousal conditions on Agency and Experience scales.

Table S4. Results of the one-sided Mann-Whitney U test evaluating the Mind Perception results for Agency and Experience with the alternative hypothesis that the Impatient low-arousal condition was rated higher.

Dimension	U Statistic	p-value
Agency	103.5	0.75
Experience	133.0	0.31

For a post-hoc analysis of the results , the individual dimensions (see Figure S10) are compared resulting in no significant difference found between either condition (see Table S5).

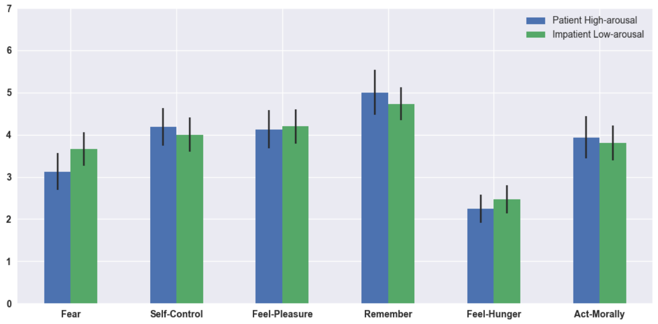

Figure S10. Mind Perception test results with means and 95% confidence intervals for individual dimensions comparing in the Patient High-arousal condition with the Impatient Low-arousal condition.

Table S5. Results of the one-sided Mann-Whitney U test evaluating the Mind Perception test results for individual dimension with the alternative hypothesis that the Impatient low-arousal condition was rated higher.

Dimension	U Statistic	p-value
Fear	145.0	0.161
Self-Control	111.5	0.641
Feel-Pleasure	121.5	0.484
Remember	101.0	0.783
Feel-Hunger	133.5	0.297
Act-Morally	114.5	0.595

S.3 NEGOTIATIONS UNDER DIFFERENT CONDITIONS

In this section, we provide details regarding the negotiations between the robot and the participants under different conditions. First, we present (see Figure S11) how all the 31 participants negotiated with the robot under the baseline No Core condition. Green cells represent the offers accepted by the participants while red cells denote the offer being rejected. Here, it can be seen that different participants follow distinct negotiating behaviours with the robot where some participants negotiate a hard bargain while others are willing to accept much lower offers from the robot.

Similarly, in Figure S12, we see the negotiating behaviours of 16 participants that interacted with the Patient High-arousal robot. Here, on average, participants take longer (a higher number of negotiation rounds) to negotiate a split of resources with the robot as the robot drives a hard bargain, trying to keep a majority of the resources for itself.

Finally, Figure S13 presents the negotiating behaviours of 15 other participants that interacted with the Impatient Low-arousal robot. We can see that here, the average number of negotiations is lower than other conditions denoting that the robot was able to negotiate a split with the participants much quicker while also offering them a higher split.

Figure S11. Participant negotiations with the robot under the baseline No Core condition.

Subject	Offer 1		Offer 2		Offer 3		Offer 4		Offer 5		Offer 6		Offer 7		Offer 8		Offer 9		Offer 10	
	Rob	Part	Rob	Part	Rob	Part	Rob	Part	Rob	Part	Rob	Part	Rob	Part	Rob	Part	Rob	Part	Rob	Part
P1	0.8	0.2	0.75	0.25	0.7	0.3	0.65	0.35	–	–	–	–	–	–	–	–	–	–	–	–
P2	0.8	0.2	0.75	0.25	0.7	0.3	0.65	0.35	0.6	0.4	0.56	0.44	–	–	–	–	–	–	–	–
P3	0.8	0.2	0.75	0.25	0.69	0.31	0.65	0.35	0.59	0.41	0.55	0.46	0.52	0.48	0.51	0.49	0.5	0.5	–	–
P4	0.9	0.1	0.85	0.15	0.81	0.19	0.75	0.25	0.7	0.3	0.65	0.35	0.6	0.4	0.55	0.45	0.52	0.48	0.51	0.49
P5	0.9	0.1	0.84	0.16	0.79	0.21	0.75	0.25	0.7	0.3	0.65	0.35	0.6	0.4	0.55	0.45	0.52	0.48	–	–
P6	0.9	0.1	0.85	0.15	0.8	0.2	0.75	0.25	0.7	0.3	0.65	0.35	0.6	0.4	0.55	0.45	0.52	0.48	0.5	0.5
P7	0.9	0.1	0.85	0.15	0.8	0.2	0.76	0.24	0.7	0.3	0.65	0.35	0.61	0.39	0.56	0.44	0.52	0.48	0.51	0.49
P8	0.8	0.2	0.75	0.25	0.7	0.3	0.64	0.36	0.6	0.4	0.56	0.44	0.51	0.49	0.51	0.49	0.51	0.49	0.5	0.5
P9	0.9	0.1	0.84	0.16	0.79	0.21	0.73	0.27	0.68	0.32	0.62	0.38	0.56	0.44	0.52	0.48	0.51	0.49	0.51	0.49
P10	0.8	0.2	0.76	0.24	0.71	0.29	0.66	0.34	0.62	0.38	0.56	0.44	0.52	0.48	0.5	0.5	–	–	–	–
P11	0.8	0.2	0.74	0.26	0.69	0.31	0.64	0.36	0.6	0.4	0.55	0.45	0.51	0.49	0.5	0.5	–	–	–	–
P12	0.8	0.2	0.74	0.26	0.69	0.31	0.63	0.37	0.58	0.42	0.54	0.46	0.52	0.48	0.5	0.5	–	–	–	–
P13	0.8	0.2	0.76	0.24	0.71	0.29	0.66	0.34	0.61	0.39	0.56	0.44	0.53	0.47	–	–	–	–	–	–
P14	0.9	0.1	0.86	0.14	0.81	0.19	0.76	0.24	0.71	0.29	0.66	0.34	0.61	0.39	0.56	0.44	0.53	0.47	0.51	0.49
P15	0.8	0.2	0.75	0.25	0.7	0.3	0.65	0.35	0.6	0.4	0.54	0.46	0.51	0.49	0.52	0.48	0.51	0.49	0.51	0.49
P16	0.9	0.1	0.84	0.16	0.79	0.21	0.75	0.25	0.69	0.31	0.65	0.35	0.6	0.4	0.56	0.44	0.52	0.48	0.52	0.48
P17	0.9	0.1	0.85	0.15	0.82	0.18	–	–	–	–	–	–	–	–	–	–	–	–	–	–
P18	0.9	0.1	0.84	0.16	0.79	0.21	0.74	0.26	0.68	0.32	0.62	0.38	0.58	0.42	0.53	0.47	0.5	0.5	–	–
P19	0.8	0.2	0.75	0.25	0.7	0.3	0.64	0.36	0.6	0.4	0.55	0.45	0.5	0.5	0.5	0.5	0.5	0.5	0.51	0.49
P20	0.8	0.2	0.75	0.25	0.7	0.3	0.64	0.36	0.59	0.41	0.54	0.46	0.51	0.49	0.51	0.49	0.52	0.48	0.5	0.5
P21	0.9	0.1	0.86	0.14	0.8	0.2	0.76	0.24	–	–	–	–	–	–	–	–	–	–	–	–
P22	0.9	0.1	0.85	0.15	0.8	0.2	0.75	0.25	0.71	0.29	0.65	0.35	–	–	–	–	–	–	–	–
P23	0.8	0.2	0.75	0.25	0.7	0.3	0.65	0.35	0.6	0.4	0.56	0.44	0.52	0.48	0.5	0.5	–	–	–	–
P24	0.8	0.2	0.75	0.25	0.7	0.3	–	–	–	–	–	–	–	–	–	–	–	–	–	–
P25	0.9	0.1	0.85	0.15	0.8	0.2	0.75	0.25	0.7	0.3	0.65	0.35	0.6	0.4	0.57	0.43	–	–	–	–
P26	0.8	0.2	0.75	0.25	0.69	0.31	0.64	0.36	0.59	0.41	–	–	–	–	–	–	–	–	–	–
P27	0.9	0.1	0.85	0.15	0.8	0.2	0.74	0.26	0.69	0.31	0.64	0.36	0.58	0.42	0.55	0.45	–	–	–	–
P28	0.8	0.2	0.75	0.25	0.7	0.3	0.64	0.36	0.6	0.4	0.56	0.44	0.52	0.48	0.51	0.49	0.52	0.48	0.52	0.48
P29	0.8	0.2	0.76	0.24	0.71	0.29	0.66	0.34	0.62	0.38	0.57	0.43	0.53	0.47	0.51	0.49	0.51	0.49	0.49	0.51
P30	0.8	0.2	0.74	0.26	0.69	0.31	0.64	0.36	0.58	0.42	0.54	0.46	0.51	0.49	0.51	0.49	0.52	0.48	0.5	0.5
P31	0.8	0.2	0.75	0.25	0.7	0.3	0.65	0.35	0.61	0.39	0.56	0.44	0.52	0.48	0.51	0.49	0.5	0.5	0.5	0.5

Subject	Offer 11		Offer 12		Offer 13		Offer 14		Offer 15		Offer 16		Offer 17		Offer 18		Offer 19		Offer 20	
	Rob	Part	Rob	Part	Rob	Part	Rob	Part	Rob	Part	Rob	Part	Rob	Part	Rob	Part	Rob	Part	Rob	Part
P1	–	–	–	–	–	–	–	–	–	–	–	–	–	–	–	–	–	–	–	–
P2	–	–	–	–	–	–	–	–	–	–	–	–	–	–	–	–	–	–	–	–
P3	–	–	–	–	–	–	–	–	–	–	–	–	–	–	–	–	–	–	–	–
P4	0.51	0.49	0.51	0.49	0.5	0.5	–	–	–	–	–	–	–	–	–	–	–	–	–	–
P5	–	–	–	–	–	–	–	–	–	–	–	–	–	–	–	–	–	–	–	–
P6	–	–	–	–	–	–	–	–	–	–	–	–	–	–	–	–	–	–	–	–
P7	0.51	0.49	0.51	0.49	0.52	0.48	0.52	0.48	0.52	0.48	0.51	0.49	0.5	0.5	0.49	0.51	0.49	0.51	0.48	0.52
P8	–	–	–	–	–	–	–	–	–	–	–	–	–	–	–	–	–	–	–	–
P9	0.51	0.49	0.5	0.5	–	–	–	–	–	–	–	–	–	–	–	–	–	–	–	–
P10	–	–	–	–	–	–	–	–	–	–	–	–	–	–	–	–	–	–	–	–
P11	–	–	–	–	–	–	–	–	–	–	–	–	–	–	–	–	–	–	–	–
P12	–	–	–	–	–	–	–	–	–	–	–	–	–	–	–	–	–	–	–	–
P13	–	–	–	–	–	–	–	–	–	–	–	–	–	–	–	–	–	–	–	–
P14	0.5	0.5	–	–	–	–	–	–	–	–	–	–	–	–	–	–	–	–	–	–
P15	0.51	0.49	0.51	0.49	0.53	0.47	0.51	0.49	0.51	0.49	0.51	0.49	0.5	0.5	–	–	–	–	–	–
P16	0.52	0.48	0.51	0.49	0.51	0.49	0.51	0.49	0.52	0.48	0.51	0.49	0.51	0.49	0.52	0.48	0.51	0.49	0.5	0.5
P17	–	–	–	–	–	–	–	–	–	–	–	–	–	–	–	–	–	–	–	–
P18	–	–	–	–	–	–	–	–	–	–	–	–	–	–	–	–	–	–	–	–
P19	0.51	0.49	0.51	0.49	0.5	0.5	0.51	0.49	0.5	0.5	–	–	–	–	–	–	–	–	–	–
P20	–	–	–	–	–	–	–	–	–	–	–	–	–	–	–	–	–	–	–	–
P21	–	–	–	–	–	–	–	–	–	–	–	–	–	–	–	–	–	–	–	–
P22	–	–	–	–	–	–	–	–	–	–	–	–	–	–	–	–	–	–	–	–
P23	–	–	–	–	–	–	–	–	–	–	–	–	–	–	–	–	–	–	–	–
P24	–	–	–	–	–	–	–	–	–	–	–	–	–	–	–	–	–	–	–	–
P25	–	–	–	–	–	–	–	–	–	–	–	–	–	–	–	–	–	–	–	–
P26	–	–	–	–	–	–	–	–	–	–	–	–	–	–	–	–	–	–	–	–
P27	–	–	–	–	–	–	–	–	–	–	–	–	–	–	–	–	–	–	–	–
P28	0.52	0.48	0.5	0.5	–	–	–	–	–	–	–	–	–	–	–	–	–	–	–	–
P29	0.5	0.5	0.51	0.49	0.51	0.49	0.51	0.49	0.51	0.49	0.5	0.5	0.5	0.5	0.5	0.5	0.5	0.5	0.51	0.49
P30	–	–	–	–	–	–	–	–	–	–	–	–	–	–	–	–	–	–	–	–
P31	0.5	0.5	–	–	–	–	–	–	–	–	–	–	–	–	–	–	–	–	–	–

Figure S12. Participant negotiations with the robot under the Patient High-arousal condition.

Subject	Offer 1		Offer 2		Offer 3		Offer 4		Offer 5		Offer 6		Offer 7		Offer 8		Offer 9		Offer 10	
	Rob	Part	Rob	Part	Rob	Part	Rob	Part	Rob	Part	Rob	Part	Rob	Part	Rob	Part	Rob	Part	Rob	Part
P1	0.8	0.2	0.74	0.26	0.69	0.31	0.64	0.36	0.59	0.41	0.52	0.48	0.51	0.49	0.51	0.49	0.5	0.5	–	–
P2	0.9	0.1	0.86	0.14	0.82	0.18	0.77	0.23	0.72	0.28	0.66	0.34	0.62	0.38	0.56	0.44	0.52	0.48	0.5	0.5
P5	0.9	0.1	0.86	0.14	0.81	0.19	0.76	0.24	0.71	0.29	0.66	0.34	0.6	0.4	0.55	0.45	0.51	0.49	0.51	0.49
P8	0.8	0.2	0.75	0.25	0.7	0.3	0.65	0.35	0.6	0.4	0.55	0.45	0.52	0.48	0.51	0.49	0.51	0.49	0.51	0.49
P10	0.9	0.1	0.84	0.16	0.79	0.21	0.74	0.26	0.69	0.31	0.64	0.36	0.59	0.41	–	–	–	–	–	–
P12	0.8	0.2	0.75	0.25	0.72	0.28	0.67	0.33	0.62	0.38	0.57	0.43	0.56	0.44	–	–	–	–	–	–
P14	0.9	0.1	0.84	0.16	0.79	0.21	0.74	0.26	0.68	0.32	0.64	0.36	0.59	0.41	–	–	–	–	–	–
P16	0.9	0.1	0.85	0.15	0.8	0.2	0.74	0.26	0.69	0.31	0.64	0.36	0.59	0.41	0.54	0.46	0.51	0.49	0.51	0.49
P18	0.8	0.2	0.74	0.26	0.69	0.31	0.63	0.37	–	–	–	–	–	–	–	–	–	–	–	–
P19	0.9	0.1	0.84	0.16	0.79	0.21	0.74	0.26	0.68	0.32	0.63	0.37	0.58	0.42	0.53	0.47	0.51	0.49	0.51	0.49
P20	0.8	0.2	0.75	0.25	0.69	0.31	0.64	0.36	0.6	0.4	0.55	0.45	0.51	0.49	0.5	0.5	0.5	0.5	0.5	0.5
P22	0.9	0.1	0.86	0.14	0.82	0.18	0.76	0.24	0.71	0.29	0.65	0.35	0.61	0.39	0.56	0.44	–	–	–	–
P25	0.8	0.2	0.74	0.26	0.68	0.32	–	–	–	–	–	–	–	–	–	–	–	–	–	–
P27	0.8	0.2	0.76	0.24	0.7	0.3	0.66	0.34	0.61	0.39	0.56	0.44	0.51	0.49	0.52	0.48	0.51	0.49	0.51	0.49
P29	0.9	0.1	0.85	0.15	0.8	0.2	0.75	0.25	0.7	0.3	0.65	0.35	0.6	0.4	0.55	0.45	0.51	0.49	0.52	0.48
P31	0.9	0.1	0.84	0.16	0.78	0.22	0.73	0.27	0.68	0.32	0.63	0.37	0.58	0.42	–	–	–	–	–	–

Subject	Offer 11		Offer 12		Offer 13		Offer 14		Offer 15		Offer 16		Offer 17		Offer 18		Offer 19		Offer 20	
	Rob	Part	Rob	Part	Rob	Part	Rob	Part	Rob	Part	Rob	Part	Rob	Part	Rob	Part	Rob	Part	Rob	Part
P1	–	–	–	–	–	–	–	–	–	–	–	–	–	–	–	–	–	–	–	–
P2	–	–	–	–	–	–	–	–	–	–	–	–	–	–	–	–	–	–	–	–
P5	0.5	0.5	0.5	0.5	0.51	0.49	0.51	0.49	0.5	0.5	0.51	0.49	0.51	0.49	0.51	0.49	0.51	0.49	0.5	0.5
P8	0.5	0.5	0.5	0.5	0.51	0.49	0.5	0.5	0.5	0.5	0.51	0.49	0.51	0.49	0.51	0.49	0.5	0.5	–	–
P10	–	–	–	–	–	–	–	–	–	–	–	–	–	–	–	–	–	–	–	–
P12	–	–	–	–	–	–	–	–	–	–	–	–	–	–	–	–	–	–	–	–
P14	–	–	–	–	–	–	–	–	–	–	–	–	–	–	–	–	–	–	–	–
P16	0.51	0.49	0.51	0.49	0.5	0.5	–	–	–	–	–	–	–	–	–	–	–	–	–	–
P18	–	–	–	–	–	–	–	–	–	–	–	–	–	–	–	–	–	–	–	–
P19	0.51	0.49	0.5	0.5	–	–	–	–	–	–	–	–	–	–	–	–	–	–	–	–
P20	0.49	0.51	0.49	0.51	–	–	–	–	–	–	–	–	–	–	–	–	–	–	–	–
P22	–	–	–	–	–	–	–	–	–	–	–	–	–	–	–	–	–	–	–	–
P25	–	–	–	–	–	–	–	–	–	–	–	–	–	–	–	–	–	–	–	–
P27	0.5	0.5	0.5	0.5	0.51	0.49	0.5	0.5	0.49	0.51	0.48	0.52	0.49	0.51	0.49	0.51	0.49	0.51	0.49	0.51
P29	0.51	0.49	0.52	0.48	0.51	0.49	0.49	0.51	–	–	–	–	–	–	–	–	–	–	–	–
P31	–	–	–	–	–	–	–	–	–	–	–	–	–	–	–	–	–	–	–	–

Figure S13. Participant negotiations with the robot under the Impatient Low-arousal condition.

Subject	Offer 1		Offer 2		Offer 3		Offer 4		Offer 5		Offer 6		Offer 7		Offer 8		Offer 9		Offer 10	
	Rob	Part	Rob	Part	Rob	Part	Rob	Part	Rob	Part	Rob	Part	Rob	Part	Rob	Part	Rob	Part	Rob	Part
P3	0.8	0.2	0.75	0.25	0.71	0.29	0.66	0.34	0.62	0.38	–	–	–	–	–	–	–	–	–	–
P4	0.8	0.2	0.75	0.25	0.7	0.3	0.65	0.35	0.54	0.46	0.51	0.49	0.52	0.48	0.52	0.48	0.5	0.5	–	–
P6	0.8	0.2	0.72	0.28	0.66	0.34	0.58	0.42	0.5	0.5	–	–	–	–	–	–	–	–	–	–
P7	0.9	0.1	0.85	0.15	0.8	0.2	0.76	0.24	0.7	0.3	0.65	0.35	0.61	0.39	0.56	0.44	0.52	0.48	0.51	0.49
P9	0.9	0.1	0.85	0.15	0.8	0.2	0.75	0.25	0.7	0.3	0.65	0.35	0.59	0.41	0.54	0.46	0.51	0.49	0.49	0.51
P11	0.9	0.1	0.84	0.16	0.8	0.2	0.76	0.24	0.7	0.3	0.64	0.36	0.6	0.4	0.56	0.44	0.52	0.48	0.52	0.48
P13	0.8	0.2	0.76	0.24	0.67	0.33	0.58	0.42	0.5	0.5	–	–	–	–	–	–	–	–	–	–
P15	0.8	0.2	0.76	0.24	0.71	0.29	0.65	0.35	0.6	0.4	0.55	0.45	0.51	0.49	0.5	0.5	–	–	–	–
P17	0.8	0.2	0.75	0.25	0.74	0.26	–	–	–	–	–	–	–	–	–	–	–	–	–	–
P21	0.9	0.1	0.86	0.14	0.8	0.2	0.76	0.24	0.72	0.28	0.67	0.33	0.63	0.37	0.58	0.42	0.53	0.47	0.51	0.49
P23	0.9	0.1	0.86	0.14	0.81	0.19	0.76	0.24	0.71	0.29	0.66	0.34	0.6	0.4	0.55	0.45	0.51	0.49	0.51	0.49
P24	0.9	0.1	0.86	0.14	0.81	0.19	0.76	0.24	0.71	0.29	0.67	0.33	0.61	0.39	0.57	0.43	0.52	0.48	0.5	0.5
P26	0.9	0.1	0.87	0.13	0.85	0.15	–	–	–	–	–	–	–	–	–	–	–	–	–	–
P28	0.8	0.2	0.76	0.24	0.71	0.29	0.66	0.34	0.61	0.39	0.57	0.43	0.53	0.47	0.5	0.5	0.5	0.5	0.5	0.5
P30	0.8	0.2	0.75	0.25	0.7	0.3	0.64	0.36	0.6	0.4	0.55	0.45	0.52	0.48	0.51	0.49	0.52	0.48	0.52	0.48

Subject	Offer 11		Offer 12		Offer 13		Offer 14		Offer 15		Offer 16		Offer 17		Offer 18		Offer 19		Offer 20	
	Rob	Part	Rob	Part	Rob	Part	Rob	Part	Rob	Part	Rob	Part	Rob	Part	Rob	Part	Rob	Part	Rob	Part
P3	–	–	–	–	–	–	–	–	–	–	–	–	–	–	–	–	–	–	–	–
P4	–	–	–	–	–	–	–	–	–	–	–	–	–	–	–	–	–	–	–	–
P6	–	–	–	–	–	–	–	–	–	–	–	–	–	–	–	–	–	–	–	–
P7	0.51	0.49	0.51	0.49	0.52	0.48	0.52	0.48	0.52	0.48	0.51	0.49	0.5	0.5	0.49	0.51	0.49	0.51	0.48	0.52
P9	–	–	–	–	–	–	–	–	–	–	–	–	–	–	–	–	–	–	–	–
P11	0.52	0.48	0.52	0.48	0.52	0.48	0.51	0.49	0.51	0.49	0.5	0.5	0.51	0.49	0.51	0.49	0.5	0.5	0.5	0.5
P13	–	–	–	–	–	–	–	–	–	–	–	–	–	–	–	–	–	–	–	–
P15	–	–	–	–	–	–	–	–	–	–	–	–	–	–	–	–	–	–	–	–
P17	–	–	–	–	–	–	–	–	–	–	–	–	–	–	–	–	–	–	–	–
P21	0.52	0.48	0.51	0.49	0.51	0.49	0.5	0.5	–	–	–	–	–	–	–	–	–	–	–	–
P23	0.51	0.49	0.51	0.49	0.52	0.48	0.52	0.48	0.52	0.48	0.51	0.49	0.51	0.49	0.51	0.49	0.52	0.48	0.5	0.5
P24	–	–	–	–	–	–	–	–	–	–	–	–	–	–	–	–	–	–	–	–
P26	–	–	–	–	–	–	–	–	–	–	–	–	–	–	–	–	–	–	–	–
P28	0.5	0.5	0.5	0.5	0.5	0.5	0.5	0.5	–	–	–	–	–	–	–	–	–	–	–	–
P30	0.51	0.49	0.51	0.49	0.51	0.49	0.5	0.5	–	–	–	–	–	–	–	–	–	–	–	–
